# AAA+ ATPase p97/VCP mutants and inhibitor binding disrupt inter-domain coupling and subsequent allosteric activation

**DOI:** 10.1016/j.jbc.2021.101187

**Published:** 2021-09-11

**Authors:** Brian Caffrey, Xing Zhu, Alison Berezuk, Katharine Tuttle, Sagar Chittori, Sriram Subramaniam

**Affiliations:** Department of Biochemistry and Molecular Biology, University of British Columbia, Vancouver, British Columbia, Canada

**Keywords:** p97 mutants, Protein Degradation, Protein Quality Control, AAA+ ATPase, p97, VCP, CB-5083, ALS, amyotrophic lateral sclerosis, FTD, frontotemporal dementia, MSP-1, multisystem proteinopathy 1, PDB, Paget's disease of the bone

## Abstract

The human AAA+ ATPase p97, also known as valosin-containing protein, a potential target for cancer therapeutics, plays a vital role in the clearing of misfolded proteins. p97 dysfunction is also known to play a crucial role in several neurodegenerative disorders, such as MultiSystem Proteinopathy 1 (MSP-1) and Familial Amyotrophic Lateral Sclerosis (ALS). However, the structural basis of its role in such diseases remains elusive. Here, we present cryo-EM structural analyses of four disease mutants p97^R155H^, p97^R191Q^, p97^A232E^, p97^D592N^, as well as p97^E470D^, implicated in resistance to the drug CB-5083, a potent p97 inhibitor. Our cryo-EM structures demonstrate that these mutations affect nucleotide-driven allosteric activation across the three principal p97 domains (N, D1, and D2) by predominantly interfering with either (1) the coupling between the D1 and N-terminal domains (p97^R155H^ and p97^R191Q^), (2) the interprotomer interactions (p97^A232E^), or (3) the coupling between D1 and D2 nucleotide domains (p97^D592N^, p97^E470D^). We also show that binding of the competitive inhibitor, CB-5083, to the D2 domain prevents conformational changes similar to those seen for mutations that affect coupling between the D1 and D2 domains. Our studies enable tracing of the path of allosteric activation across p97 and establish a common mechanistic link between active site inhibition and defects in allosteric activation by disease-causing mutations and have potential implications for the design of novel allosteric compounds that can modulate p97 function.

p97 is a member of the classic clade of AAA+ ATPases and is an essential cellular enzyme. p97 is composed of six protomers forming a homo-hexamer with an N-terminal domain (NTD) and two tandem ATPase domains D1 and D2 (1). p97 is an essential protein in regulating cellular homeostasis from membrane fusion, Endoplasmic Reticulum-Associated Degradation, Mitochondrial-Associated Degradation (MAD), Chromatin-Associated Degradation to NF-κB activation (2). A common thread among these applications is the recruitment of numerous cofactors to process ubiquitinated substrates, through the generation of mechanical energy from ATP hydrolysis. Critical functions for p97 include the translocation and restructuring of proteins from large cellular structures such as organelle membranes and extraction of ubiquitinated client proteins to facilitate their degradation through the ubiquitin-proteasome system. In this role, p97 recruits ubiquitin-binding cofactors to denature ubiquitinated substrates by pulling the polypeptide through the central pore (3, 4).

Cryo-EM analyses of full-length p97 in the substrate-free form have established the overall organization of N, D1, and D2 domains (Fig. 1*A*) in three distinct quaternary conformations that correspond to distinct nucleotide occupancies of the D1 and D2 nucleotide-binding sites (Fig. 1*B*) (5). Conformation I is observed when ADP is bound to both the D1 and D2 domains, while conformation II is observed when ADP in the D2-binding site is exchanged with ATPγS, which is a slowly hydrolyzable ATP analog to preserve the ATP-bound structure for cryo-EM analysis. Relative to conformation I, there is a rotational twist of the D2 domain with relatively minimal changes in the D1 and N domains. Conformation III is observed when ATPγS is bound to both D1 and D2 nucleotide-binding sites and results in substantial tertiary and quaternary structural rearrangements in the D1 and N domains. The N-domain in conformation III adopts a distinct “up” position, enabling its binding to endogenous cofactors required for subsequent substrate binding and processing. The nucleotide occupancy in these three conformational states is consistent with the higher affinity of the D1 domain for both ADP and ATP ligands relative to the D2 domain, with a ∼40-fold lower K_D_ in D1 relative to D2, as measured by Surface Plasmon Resonance (SPR) experiments (6). All three conformations of substrate-free p97 display 6-fold symmetry. However, substrate binding results in the conversion of the hexameric arrangement to a spiral arrangement of the six protomers similar to the quaternary conformation observed for most other members of the AAA+ ATPase family (3, 4).

**Figure 1:**
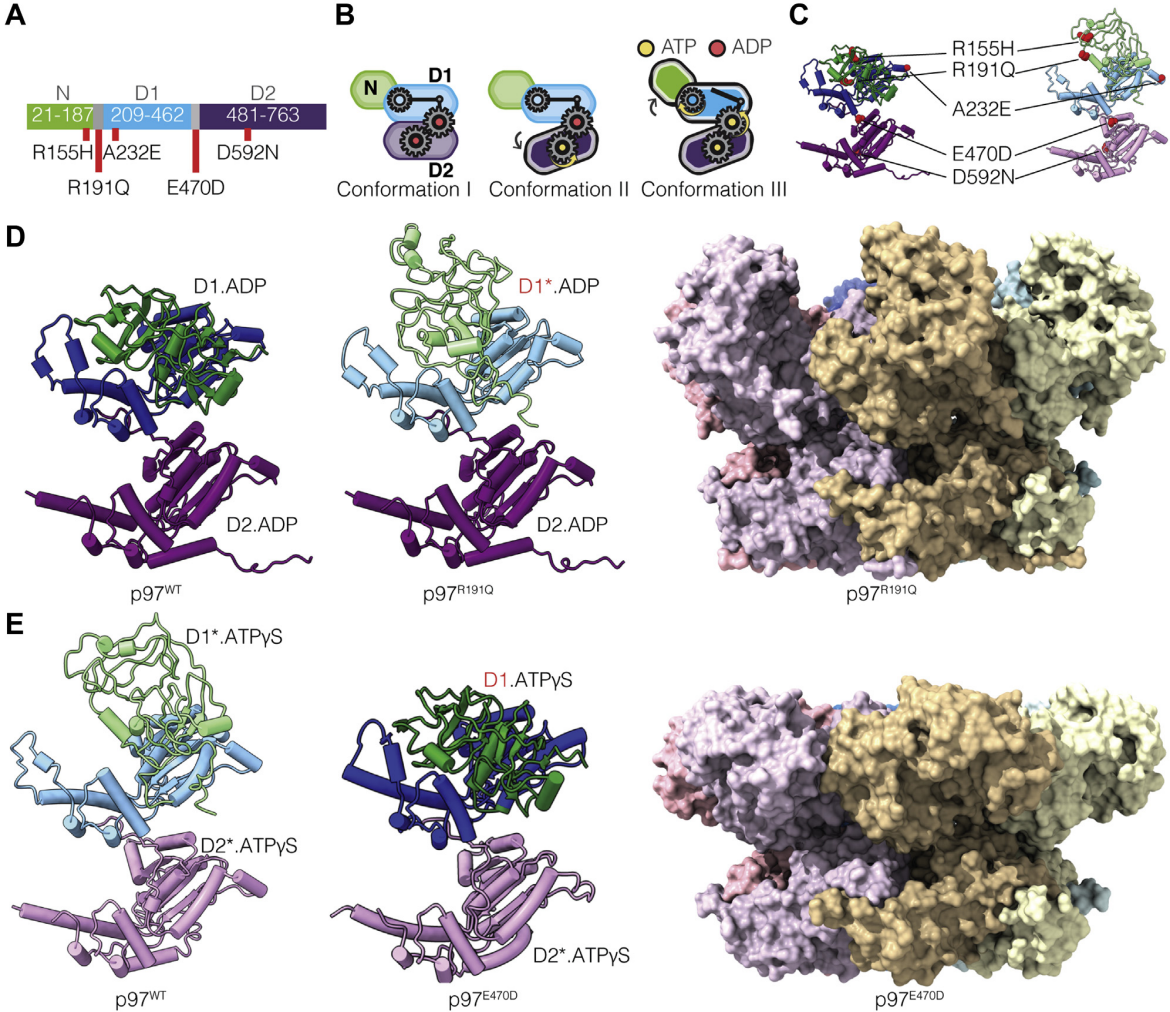
**Cryo-EM structures of p97 disease mutants**. *A*, schematic of human p97 sequence identifying the location of the three p97 domains and locations of the mutations analyzed in this study. *B*, schematic representation of the three conformations observed for p97^WT^, *arrows* indicate domain movement in p97 monomers upon nucleotide binding, with the gears illustrating interdomain communication. *C*, location of mutations labeled in (*A*) in the 3D structure of a p97^WT^ protomer in ADP-bound (*left panel*, PDB: 5FTL) and ATPγS-bound (*right panel*, PDB: 5FTN) states. *D*, atomic models for the ADP-bound p97^WT^ and p97^R191Q^ protomers (*left*) and space-filling model of the p97^R191Q^ hexamer in the ADP-bound state (*right*). *E*, atomic models for the ATPγS-bound p97^WT^ and p97^E470D^ protomers (*left*) and space-filling model of the p97^E470D^ hexamer in the ATPγS-bound state (*right*). The darker and lighter colors of the D1 and D2 domains in this and subsequent figures reflect the conformation of the domains observed in p97^WT^. For example, as shown in (*D*), the conformation of the ADP-bound D2 domain in p97^R191Q^ the same as that observed for p97^WT^, but that of the ADP-bound D1 domain is different, and closer to that seen for the ATPS-bound D1 domain.

Due to the essential role played by p97 in cellular processes, it is no surprise that a number of multisystem diseases are associated with mutations and dysfunctions in p97. Diseases arising from protein degradation defects (7) and DNA repair (8) such as cancer, viral infections such as the poliovirus (9), and even neurodegenerative disorders all implicate p97 as crucial in disease progression. p97 is therefore an attractive therapeutic target (10). The majority of p97 mutations are associated with a rare disease called Multi System Proteinopathies-1 (MSP-1), which is characterized by a cluster of muscular, bone, and neurological illnesses known as Inclusion Body Myopathy; Paget's Disease of the Bone (PDB); and FrontoTemporal Dementia (FTD) (11). As these mutations tend to cluster in the N- and D1-domains of the p97 protein and the majority of p97 cofactors bind the N-terminus of the protein, there is likely cofactor involvement in the mechanism of disease. These N-D1 mutants are also commonly characterized by an increase in basal ATPase activity (12) and higher substrate processing relative to wild-type p97 (13), although these effects have not been shown to be directly correlated. Specific mutations in the D2 domain of p97 have also been identified in closely related neurological illnesses such as Amyotrophic Lateral Sclerosis (ALS) (14), which tend to have less pronounced muscular and bone involvement suggesting a subtle but significant difference in the mechanism of disease. Still, other mutations in the D1-D2 linker are implicated in cancer drug resistance (15), reflecting the diverse role of p97 within the cell and its importance in different tissues. A central unanswered question in understanding the origin of the disease phenotype is whether the functional consequences can be traced to mutation-induced alterations in protein structure.

Prior structural information on p97 mutants largely comes from X-ray crystallographic studies of N-D1 truncated p97 constructs (16), NMR investigations of protein dynamics of full-length p97 mutants (17), and cryo-EM analysis of full-length p97 in complex with Ufd1-Npl4 cofactors (13, 18). Here, we have examined full-length forms of five p97 mutants: three were chosen for their relatively high incidence in the p97-related neurodegenerative disorder MSP-1 (R155H, R191Q, A232E), one for its relatively unusual position in the D2 domain in p97 and its role in familial ALS (D592N), and one for its identification in resistance to the drug CB-5083 (E470D) (15).

Our experiments with these mutants were designed to explore the extent to which the mutations influence the conformational landscape. While p97 is amenable to structural analysis in both substrate-free forms and in the presence of bound protein substrates and cofactors, we chose to study substrate-free complexes since our goal is to unravel the intrinsic effects of the mutations on quaternary structure. Previous studies have shown that the primary effect of MSP-1 mutations is in the transition between conformations (I-III) rather than directly on the hydrolytic domains (6) or indeed cofactor-bound complexes (13).

We also report cryo-EM structures of full-length wild-type p97 complexed to CB-5083 in the presence of ADP and ATPγS and describe how the binding of the inhibitor influences the overall quaternary arrangement of the N, D1, and D2 domains. Taken together, our results provide a detailed understanding of the structural mechanisms underlying the effects of the disease mutations and an explanation of the resistance of the E470D mutation to inhibition by CB-5083.

## Results

All five mutants displayed relative ATPase activities similar to previously reported biochemical studies (Figs. 1*C* and S1*A*) (12, 13, 15, 16, 19). The structural effects of the mutations on p97 structure as determined by cryo-EM studies fall into two distinct categories where the differences in behavior relative to p97^WT^ are observed in the presence of bound ADP but not ATPγS (Fig. 1*D*) or in the presence of bound ATPγS but not ADP (Fig. 1*E*). These effects are described in greater detail below.

### R155H, R191Q, and A232E mutants primarily affect the ADP-bound states

**Figure 2:**
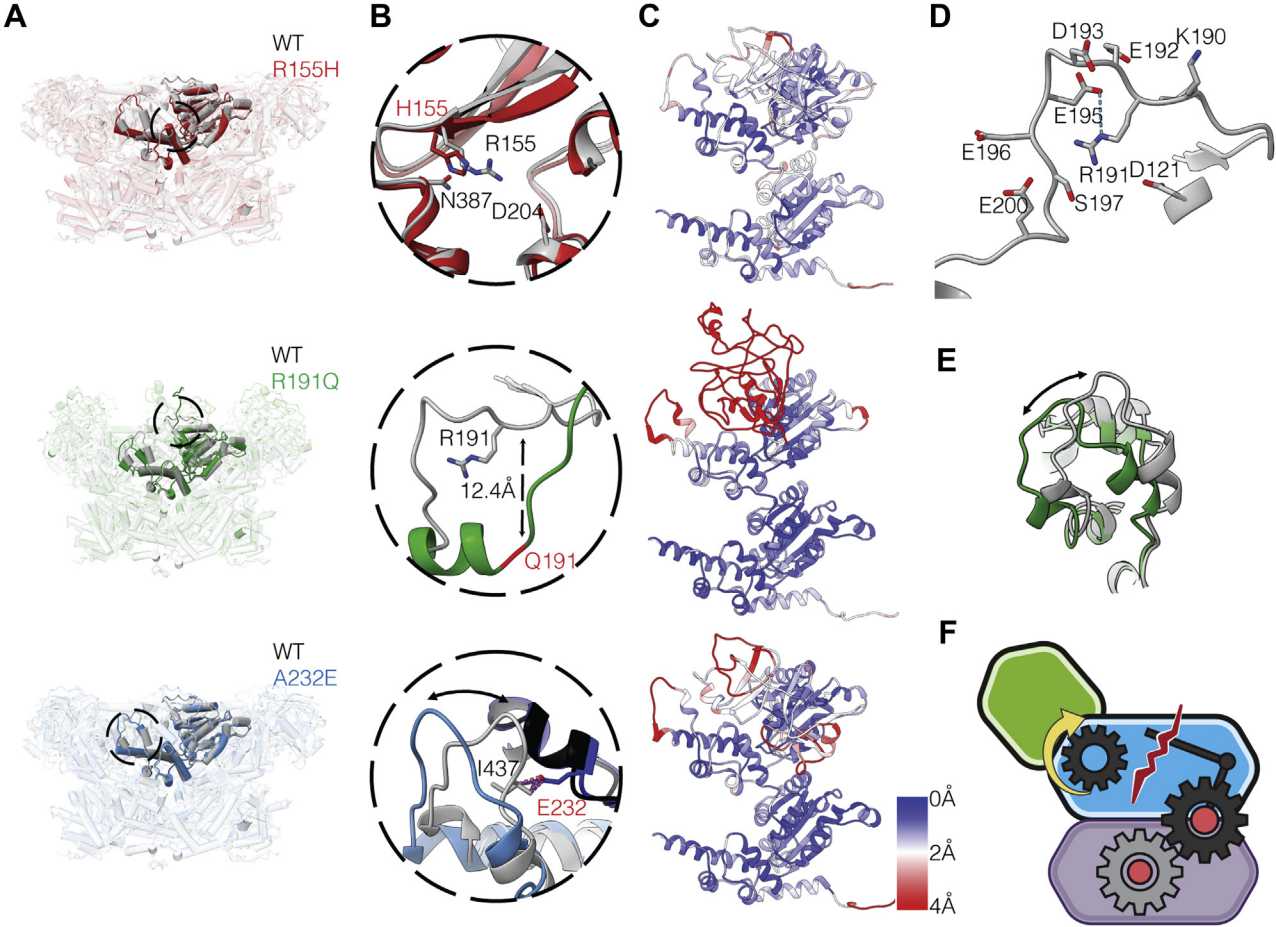
**R155H, R191Q, and A232E mutations disrupt N-D1 domain interactions**. *A*, *top*, superposition of both p97^WT^ (*gray*) and p97^R155H^ (*red*) ADP-bound hexameric structures, with the N-D1 linker and D1 domains highlighted (residues 180–460). *Middle*, superposition of both p97^WT^ (*gray*) and p97^R191Q^ (*green*) ADP-bound hexameric structures. *Bottom*, superposition of both p97^WT^ (*gray*) and p97^A232E^ (*blue*) ADP-bound hexameric structures. *B*, *top*, expanded view of the site of the p97^R155H^ mutant, illustrating an increase in the interaction between residues H155 and N387 relative to WT. *Middle*, expanded view of the site of the p97^R191Q^ mutant in the N-D1 linker region (residues 188–209), illustrating the mutant driven loop-to-helix transition. *Bottom*, steric clash between mutant E232 and adjacent hydrophobic residue in p97^WT^ structure, modeled in chimera (van der Waals overlap of ≥0.6 Å), labeled in *purple*. Individual Mutant D1-domains labeled in *light blue*/*blue* and WT D1-domains labeled in *light gray*/*black*. *C*, *top*, p97^R155H^ protomer is displayed showing backbone Root Mean Square Deviation (RMSD) from D1D2-ADP-bound p97^WT^ (PDB: 5FTK). *Middle*, p97^R191Q^ protomer is displayed showing backbone RMSD from D1D2-ADP-bound p97^WT^ (PDB: 5FTK). *Bottom*, p97^A232E^ protomer is displayed showing backbone RMSD from D1D2-ADP-bound p97^WT^ (PDB: 5FTK). *D*, N-D1 linker region of ADP-bound p97^WT^ highlighting R191 and the surrounding residues, tentative hydrogen bond modeled in chimera, according to precise geometric constraints based on (37) labeled in *cyan*. *E*, expanded view of the D1 domain loop region (residues 425–445) of p97^WT^ and p97^R191Q^. *F*, schematic illustrating mutant protomer disruption in N-D1 interface, leading to flexibility in the N-domain conformation toward the “up” position.

In the p97^R191Q^, p97^R155H^, and p97^A232E^ mutants, the primary variation in conformational outcome as compared with p97^WT^ is observed when the D1 and D2 domains are both occupied by ADP (Fig. 1*D*). In these mutants, the ADP-bound conformation of the D1 domain displays many of the structural signatures observed only upon ATPγS binding to p97^WT^, suggesting that these mutations shift the conformational equilibrium toward promoting ATP binding. This effect is most readily visible in p97^R191Q^, with p97^R155H^ and p97^A232E^ displaying similar effects in the D1 domain, but of a smaller magnitude (Figs. 2, *A*–*C*, S1, *B–D* and S2*A*).

R155 is located at the N-D1 interface in the ADP-bound state making contacts with polar residues from the N-D1 domain (20) (Fig. 2*B*). Mutation to histidine may disrupt interactions resulting in greater flexibility of the N-domain, consistent with disorder in this domain, observed as a decrease in local resolution in the N-domain cryo-EM density. This finding is consistent with previously published NMR studies where the N-domain flexibility, *i.e.*, exchange between “up” and “down” conformations in the ADP-bound state, is inversely proportional to the charged/hydrogen bonding nature of the residues at position 155 (17).

R191 is located at the start of the linker connecting the N and D1 domains (Figs. 1*A* and 2), surrounded by negatively charged residues, in particular E195 (Fig. 2, *B* and *D*). R191 may stabilize the “down” position of the N-domain by maintaining an extended loop conformation in the linker. A unique aspect of the mutation-induced structural changes in the ADP-bound state of p97^R191Q^ is a dramatic ordering of the linker connecting the N and D1 domains into an α-helical structure between residues 190–199 (Figs. 2*B* and S1*A*). This structural ordering is observed in p97^WT^ only when ATPγS is bound to the D1 domain, which arises from changes propagated at the other side of the linker proximal to the D1 nucleotide-binding site. The dramatic shift from extended linker to an α-helix upon loss of charge by mutation from arginine to glutamine appears to confirm the vital role R191 plays in stabilizing the “down” position in the N-domain of p97^WT^. Further inspection of the structure of p97^R191Q^ in the ADP-bound state shows a shift in the local conformation of the loop region between K425 and L445 of the D1 domain that is located at the junction between the N and D1 domains (Figs. 2*E* and S1*B*). The change in the structure of this loop is such that it is close to the conformation observed for this region in p97^WT^ upon the occupancy of the D1 nucleotide-binding site with ATPγS.

Residue A232 is located at the interface between two p97 protomers, and the introduction of the glutamate at this position will result in steric repulsion (Fig. 2*B*) separating the putative hydrogen bonding residues T127 and T436 of the N and D1 domains of the adjacent monomer, respectively (Fig. S1*C*). This disruption likely accounts for the increased flexibility of the N-domain and also results in a shift similar to that of p97^R191Q^ in the loop region between K425 and L445 of the D1 domain adjacent to the E232 residue (Figs. 2*B* and S1*D*). Of note, a T127A mutation has been reported in an individual with FTD (21), further suggesting the importance of this hydrogen-bonding interaction for the function of p97.

Despite varying degrees of differences in the ADP-bound state as compared with p97^WT^, all three mutants display essentially similar structures when the D1 and D2 domains are bound to ATPγS and in the state corresponding to conformation III of p97^WT^ (Fig. S3, *A–D*) (5). This is not surprising since R155, R191, and A232 are all exposed to solvent in conformation III, *i.e.*, no longer interacting at the N-D1 interface; therefore the mutations at these sites are not expected to affect the quaternary conformation significantly. The effect of the ATPγS-driven conformational change is especially noteworthy in the case of the p97^R155H^ mutant because the upward movement of the N-domain results in the displacement of R155 by ∼25 Å to a solvent exposed face of the protein, away from the N-D1 interface. In the ATPγS-bound state, p97^R155H^ and other N-D1 mutants with similar phenotypes may have an effect on cofactor or substrate binding since it is in close proximity to the site where cofactors are expected to interact with p97 (22). Indeed, the p97^R155H^ mutant has been found to have altered responses relative to p97^WT^ when bound to either p47 or p37 cofactors (23).

### E470D and D592N mutants primarily affect the ATPγS-bound states

**Figure 3:**
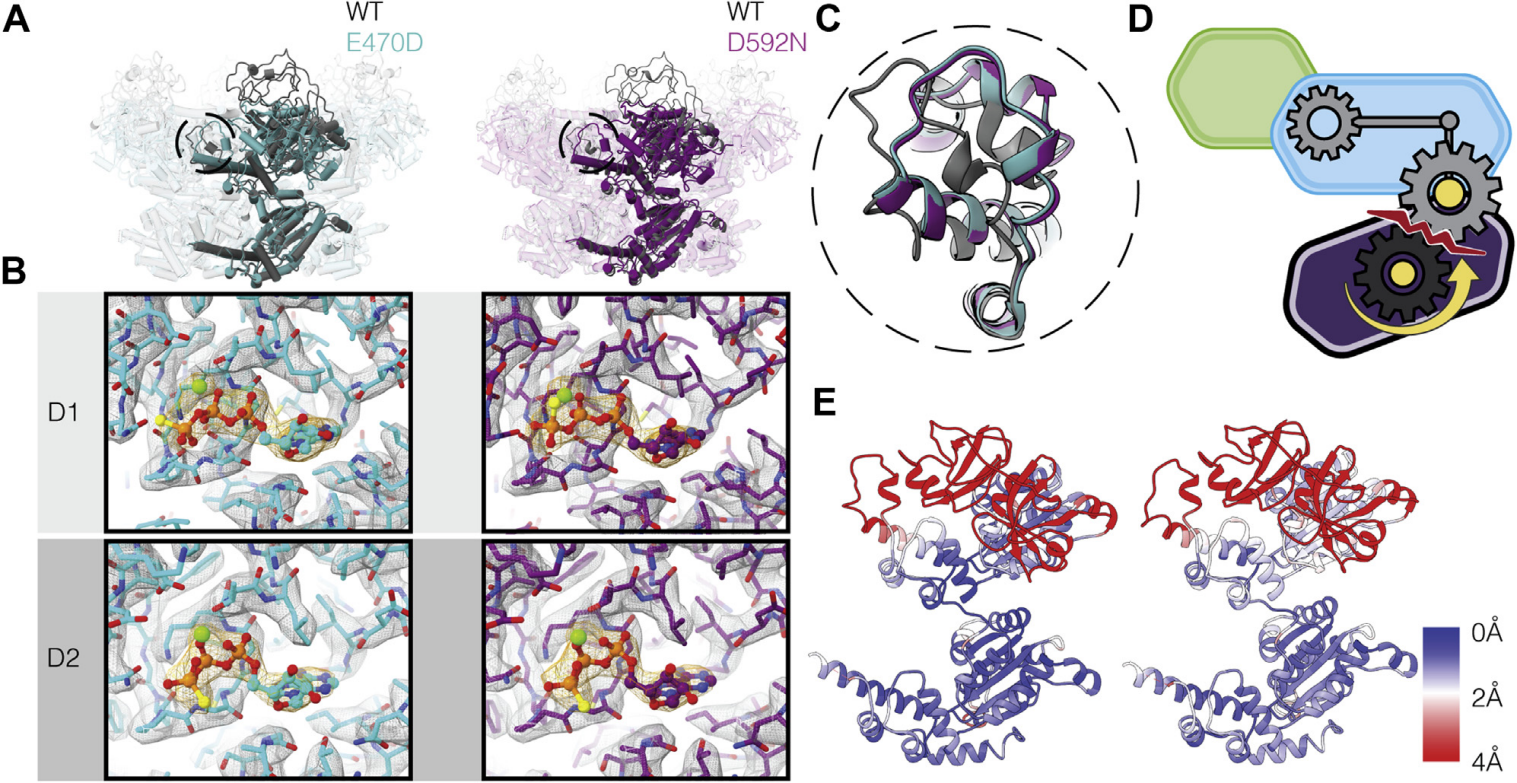
**E470D and D592N mutations alter ATP-induced conformational changes**. *A*, *left*, superposition of p97^WT^ (*gray*) and p97^E470D^ (*cyan*) ATPγS-bound hexameric structures, with monomer highlighted. *Right*, superposition of p97^WT^ (*gray*) and p97^D592N^ (*purple*) ATPγS-bound hexameric structures, with monomer highlighted. *B*, illustration of nucleotide occupancy in both the D1 and D2 domains of the unsharpened cryo-EM maps of p97^E470D^ (*left*) and p97^D592N^ (*right*) mutants, with the surrounding modeled residues, indicating the presence of ATPγS density (*orange*) in both the D1 and D2 domains. Average sigma threshold values for p97^E470D^ and p97^D592N^ EM maps are 8.4, 8.0, respectively. *C*, expanded view of the D1 domain loop region (residues 425–445) of p97^WT^ (*gray*), p97^E470D^ (*cyan*), and p97^D592N^ (*purple*). *D*, schematic showing the disruption of the intra-protomer D1–D2 communication, leading to a failure of the mutant N-domain to move into the “up” conformation upon ATPγS binding. *E*, *left*, p97^E470D^ protomer is displayed showing backbone RMSD from D1D2-ATPγS-bound p97^WT^ (PDB: 5FTN). *Right*, p97^D592N^ protomer is displayed showing backbone RMSD from D1D2-ATPγS-bound p97^WT^ (PDB: 5FTN).

In contrast to the mutants discussed above, in the p97^E470D^ and p97^D592N^ mutants, minimal structural differences are observed as compared with p97^WT^ when the D1 and D2 domains are occupied by ADP (Fig. S4). However, when bound to ATPγS, in contrast to displaying the dramatic quaternary structural changes observed in p97^WT^, the predominant conformation displayed in these two mutants is one in which the N-terminal domain is in the “down” position in a state most closely resembling conformation II of p97^WT^ (Figs. 3 and S1*E*). These results imply that the mutations decouple ATPγS binding in the D1 domain from inducing the structural rearrangement of N domain from the “down” to “up” conformation.

Although we would expect the E470D mutation to have minimal effects because of the conservative substitution, residue 470 is located at a critical juncture between the D1 and D2 domains. This major effect highlights the structural role of this side chain in maintaining the network of interactions by which the structural changes in the D2 domain are communicated to the D1 domain. D592 is located in the pore loop region of the hexamer at the inter-protomer interface (Fig. 1*C*), indicating that interactions distal to the D1-D2 interface can also influence the coupling between these domains. As observed during the activity assays, the p97^E470D^ and p97^D592N^ mutants differ in terms of their effects on ATPase activity (Fig. 1*C*). This suggests that p97^E470D^ stimulates increased ATPase activity in the D2 domain, possibly by removing the requirement for ATP binding to the D1 domain for D2 hydrolysis, whereas this intra-protomer D1-D2 communication across the linker is maintained in the p97^D592N^ mutant.

### p97 Inhibition and structural rearrangements induced by CB-5083 binding

**Figure 4:**
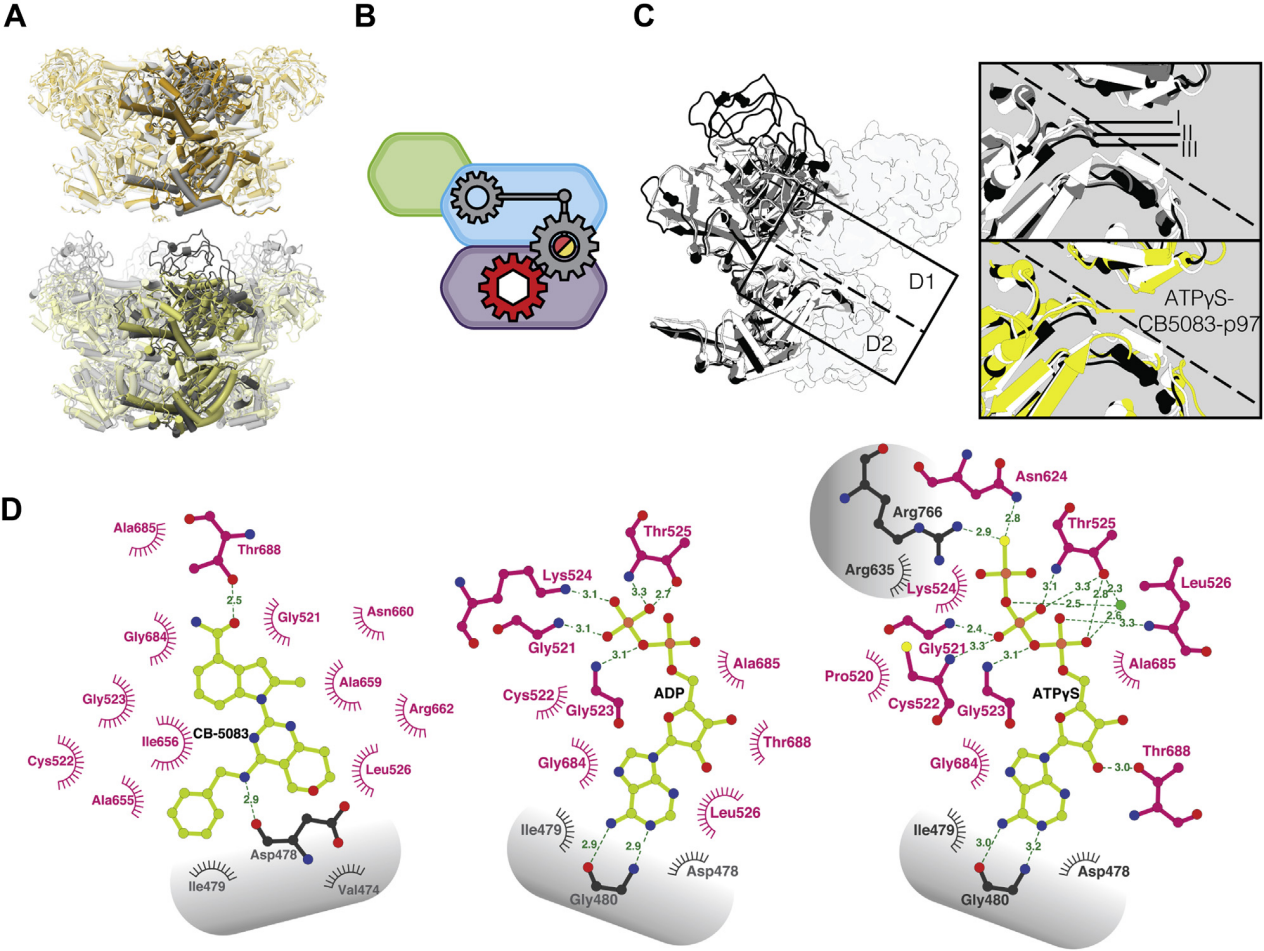
**p97 Inhibition and structural rearrangements upon CB-5083 binding**. *A*, *top*, superposition of ADP-bound p97^WT^ (*gray*) and ADP-CB-5083-bound p97^WT^ (*orange*) hexameric structures, with monomer highlighted. *Bottom*, superposition of ATPγS-bound p97^WT^ (*dark gray*) and ATPγS- CB-5083-bound p97^WT^ (*yellow*) hexameric structures, with monomer highlighted. *B*, schematic showing the complete allosteric inhibition of p97 following CB-5083 binding independent of D1 nucleotide state, illustrating D2 allosteric activation must occur before the D1 domain can proceed to the active ATPγS-bound conformation III. *C*, *left*, overlay of conformations I, II, and III of p97 monomer with adjacent monomer outlined. *Top right*, zoom into critical D1-D2 inter-protomer interactions, illustrating the key movements in the D1 and D2 domain upon sequential ATPγS-binding. *Bottom right*, overlay of the same area with conformation II omitted for clarity and the D1-ATPγS and D2-CB-5083 bound structure added in *yellow*, illustrating the absence of a conformational shift in the D2 domain leading to the inhibition of the allosteric activation of the D1 domain. *D*, Ligplot of interacting residues in the binding domain from left to right of CB5083, ADP and ATPγS. Interacting residues from adjacent protomers labeled in *gray*.

CB-5083 is a potent inhibitor of p97 function, with an IC_50_ of ∼10 nM (24). The binding site of CB-5083 to the D2 domain was characterized in a previous X-ray crystallographic study using p97 truncated to only contain the D1 and D2 domains p97^D1D2^ structure determined at a resolution of 3.75 Å (25). In order to assess the effect of CB-5083 binding in the context of full-length protein, we carried out cryo-EM studies of p97^WT^ bound to CB-5083 in the presence of either ADP or ATPγS. The cryo-EM structure of the ADP- CB-5083- p97^WT^ complex resolved at an overall resolution 2.5 Å allows unambiguous positioning of CB-5083 in the D2 domain. The inhibitor is held in place by a combination of polar interactions mediated through its terminal amide side chain and central quinazoline scaffold, and nonpolar interactions involving the pendant phenyl group. The tertiary and quaternary structures of the D1 and D2 domains remain the same under both conditions, demonstrating that when CB-5083 is bound to the D2 domain, the conformation of the D1 domain is locked and unresponsive to either the presence or the identity of the bound nucleotide. Importantly, the N-domain is in the “down” position when either ADP or ATPγS occupies the D1 nucleotide-binding site (Figs. 4*A* and S2*B*). Notably, the vast majority of p97 particles bound to CB-5083 were in the dodecameric state, regardless of D1 nucleotide occupancy (Figs. S15 and S16); this state is often observed only in the ADP-bound p97^WT^ structures but not in the ATPγS-bound structures, further supporting the observation that the CB-5083-bound D2 domain is locked in conformation I. In this respect, the effect of CB-5083 binding is similar to that observed for the E470D and D592N mutants, where the conformation of the N and D1 domains is unchanged with binding of either ADP or ATPγS (Fig. 4*B*). However, unlike these mutants, CB-5083-bound p97^WT^ is locked in conformation I and retains the same tertiary structure in the D2 domain as that seen for ADP-bound p97.

CB-5083, as an ATP-competitive inhibitor, can exert its effects on p97 function by blocking the allosteric conformational changes required for transition from conformation I to conformation II (Fig. 4*C*). The same step is blocked by the phenyl indole-based allosteric inhibitor UPCDC-30245, which binds p97 distal to the nucleotide binding site, but prevents the transition to conformation II because of a steric block of the conformational change in the D2 domain by the bound inhibitor (5).

A detailed inspection of the interactions in the nucleotide-binding pocket provides an explanation for the effects observed with CB-5083 binding. As shown in Figure 4*D*, the interactions of the two phosphate moieties in ADP are confined to a single nucleotide-binding domain, but when ATPγS is bound, the third (gamma) phosphate moiety is involved in H-bonding interactions with R766 and R635 in the neighboring protomer. These additional interactions explain why ATPγS binding initiates the sequence of quaternary structural changes *via* interactions at the inter-protomer interface. Although CB-5083 binding site overlaps with the nucleotide-binding site of the D2 domain and shares interactions with some of the residues that interact with ADP/ATP, the key inter-protomer interaction between R766 and the γ-phosphate of ATP is missing in the CB-5083-bound structure, preventing the generation of the type of quaternary conformational changes seen upon ATPγS binding to p97^WT^.

## Discussion

The cryo-EM structural studies presented here allow us to propose a structural model for the observed increase in ATPase activity of MSP-1 related p97 mutants. Our results demonstrate that the effects of the N-D1 mutations are mediated chiefly through the destabilization of the N domain in the ADP-bound state, either through a loss of interdomain interactions in p97^R155H^ and p97^R191Q^ or through inter-protomer steric effects in p97^A232E^. We expect that the primary effect of the N-D1 mutants on p97 is a disruption in cofactor recruitment *in-vivo*, due to the shift in the conformational equilibrium toward the N domain “up” state. This is supported by previous biochemical and NMR analysis (17, 26) that showed these mutants had an increased affinity for cofactors, which bound p97 preferentially in the “up” conformation, *e.g.*, p47 and Ufd1-Npl4, and a decreased affinity for co-factors, which bind p97 in the “down” conformation, namely UBXD1. These observations may therefore explain why these mutants appear to only affect a small subset of lysosome-related functions, leading to the late-onset, degenerative symptoms characteristic of the MSP-1 diseases, rather than a more global impairment of p97, where knockouts in mice were found to be lethal (27).

While the majority of both mutant and wild-type p97 ATPase activity occurs in the D2 domain (16, 28), the rate of D2 ATP hydrolysis is highly dependent on the presence of nucleotide in the D1 domain (12), suggesting a clear path of communication of nucleotide state between D1 and D2 domains. However, we have observed no significant change in the nucleotide-binding sites of the D2 domain in the N-D1 mutants. Therefore, the previously observed increase in N-D1 mutant ADP off-rate (6) and ATPase activity may not necessarily be due to a direct conformational change in the D2 nucleotide-binding domain. Instead a shift toward an ATP-like state in the D1 domain of the ADP-bound p97 may facilitate a quicker ATP turnover in the D2 domain through this interdomain communication pathway.

Analysis of the p97^E470D^ mutant structure bound to either ATPγS (Fig. 3) or CB-5083 (data not shown) in the D2 domain appears to show no significant change in the local binding pocket of either nucleotide or drug relative to WT structures, suggesting that the resistance exhibited by this mutant may not be directly related to the changes in binding affinities. Instead p97^E470D^ may exert its effects *via* changes in the ATPγS-bound conformation by decoupling the D2 domain from the D1 domain, suggesting a second possible mechanism for p97 dysfunction. This decoupling, likely through a disruption in the may lead to the observed increase in ATPase activity (15) (Fig. S1*A*), and it is possible that the requirement for nucleotide binding in the D1 domain for ATP hydrolysis in the D2 domain in WT is abolished by the E470D mutation, allowing the D2 domain to cycle through ATP, independent of D1 nucleotide state. Previously, it was shown that the D1–D2 linker residue, L464, is key to transmitting D2 nucleotide-dependent motion to the D1 domain *via* an inter-protomer pathway (29). Our studies of p97^E470D^ suggest that this residue is extremely important in maintaining the network of interactions by which the structural changes in the D2 domain are communicated to the D1 domain. This could be achieved by destabilizing the D1–D2 linker downstream, leading to inadequate transmission of D2 motion to L464, thereby blocking the critical inter-protomer communication with the adjacent 355–360 residues. While p97^D592N^ mutants appear to result in a similar disruption of ATP-induced conformational changes, this does not appear to translate to an effect on the rate of ATP hydrolysis (19). This suggests that the D2 domain, ATP-hydrolysis of p97^D592N^ may still rely on the communication of the nucleotide state of the D1 domain, further highlighting the importance of the D1-D2 linker (463–480) in regulating D2 ATP hydrolysis.

**Figure 5:**
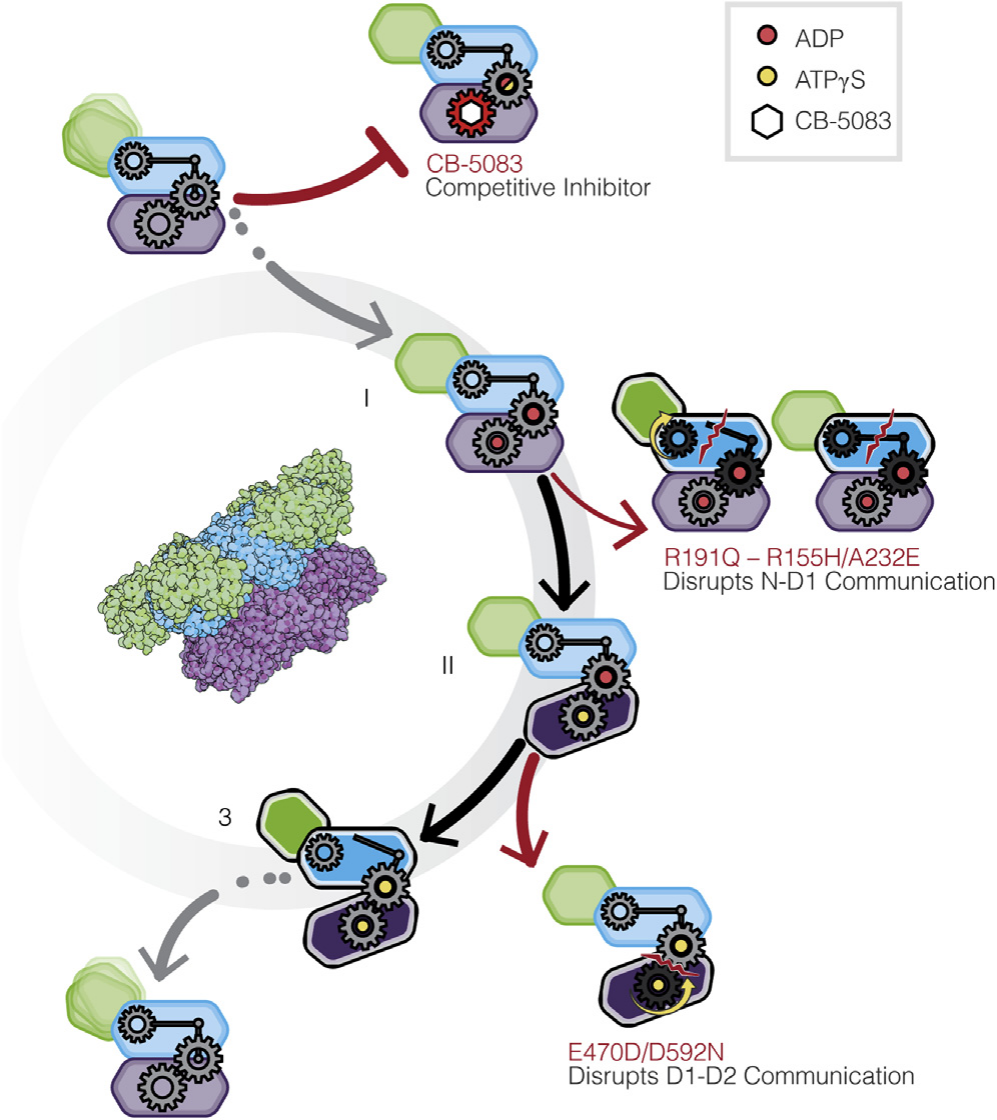
**p97 ATPase cycle.** Scheme of p97 protomer with its three major domains labeled *green*, *blue*, and *purple* (N, D1, and D2, respectively), representing the four discrete stages of nucleotide binding in p97^WT^ and the effects of mutants/inhibitors on each of these stages.

Our results indicate that interdomain communication between D1 and D2 domains can be influenced by distal interactions communicated to the domain interface *via* allosteric effects. Importantly, our structural studies with CB-5083 demonstrate that although this inhibitor competes with ATP binding to the D2 domain, the net effect of inhibitor binding is to block the critical allosteric changes that occur *via* inter-protomer and interdomain communication in a manner similar to that observed with inhibitors such as UPCDC-30245 (5) that do not block ADP binding to either D1 or D2 sites, but also lock p97 in conformation I as observed with CB-5083. That is, despite the presence of ATPγS in the D1 domain, it is necessary for the D2 domain to move into the active ATPγS-bound conformation II before the D1 domain can proceed to the N-domain “up” conformation III. This suggests that while nucleotide state communication from D1 to D2 affects D2 ATPase rate, communication in the opposite direction from D2 to D1 of nucleotide state also affects the N-domain conformation and therefore potentially cofactor recruitment. This conformational block upon inhibitor binding occurs in conformation I in contrast to what we observe in the CB-5083 resistant, p97^E470D^ mutant where the block occurs at conformation II, demonstrating that the movement from conformation I to II is necessary to proceed to conformation III, but not sufficient. Altogether, the analysis of the structures of p97 disease mutants and of the structure of p97^WT^ bound to CB-5083 provides insights into the importance of inter-protomer and interdomain interactions that govern p97 activity (Fig. 5). The availability of atomic resolution models for disease mutants and understanding of the similarities and differences in allosteric changes induced by mutations *versus* inhibitor binding provide a powerful starting point for structure-guided drug design of potent modulators whose effects can be specifically tuned for regulating p97 function.

## Experimental procedures

### Construction of expression plasmids

Multiple nucleotides were substituted at areas identified in key disease mutations: c.464G>A (R155H); c.572G>A, 573T>A (R191Q); c.695C>A (A232E); c.1410G>C (E470D); c.1774G>A (D592N) (see Table S1 for full list of primer sequences) using the Q5 Site-directed mutagenesis kit (New England Biolabs, MA, USA). Each mutant sequence was verified for insertion of the correct mutation using Sanger sequencing.

### Protein expression and purification

Full-length recombinant His6x-tagged human p97 mutants were purified with some adaptations as described in (30). Briefly, after overnight incubation at 27 °C with 0.5 mM Isopropyl β-D-1-thiogalactopyranoside (IPTG) (Fisher Scientific), *E. coli* BL21 (DE3) (Thermo Fisher) cells expressing plasmid-encoded mutant p97 were suspended in 20 ml of lysis buffer; 50 mM Tris-HCL pH 8, 300 mM NaCl, 0.5 mM β-Mercaptoethanol (BME) (Fisher Scientific), and 1× Protease Inhibitor Cocktail tablet (Thermo Fisher) and lysed. Following centrifugation, imidazole was added to the resulting supernatant to a final concentration of 20 mM and loaded onto a Ni-NTA column (Thermo Fisher) and eluted with 300 mM imidazole. The eluted protein was flash frozen in liquid nitrogen and stored in aliquots at –80 °C without glycerol.

### ATPase assays

Protein concentrations were first normalized using Pierce BCA Protein Assay kit (Thermo Fisher), a standard series was prepared with the BSA provided (2 mg/ml–25 μg/μl). Twenty-five microliter of each of the standards and unknown sample were added to a 96-well plate in triplicate, 200 μl of working reagent was added to each well, and prepared according to manufacturer's guidelines. The plate was covered and incubated at 37 °C for 30 min, the plate was cooled to room temperature (RT), and the absorbance at 562 nm was measured for each well.

The ATPase activity was examined using the ADP-Glo Kinase Assay (Promega), as previously described in (31). The protein solution was diluted to 20 nM p97 with 20 μM ATP in triplicate in 5 μl of 1× Kinase Reaction Buffer A; 40 mM Tris pH 7.5, 20 mM MgCl_2_, 0.1 mg/ml BSA, and incubated at 37 °C for 30 min. After incubation, the solutions were equilibrated to RT for 5 min and 5 μl of ADP-Glo Reagent was added to stop the reaction and consume the remaining ATP. The solution was incubated for 40 min at RT. Ten microliter of Kinase Detection Reagent was added and incubated for 30 min. The sample's luminescence was recorded using Varioskan LUX Multimode Microplate reader (Thermo Fisher). Each sample was averaged across the three measurements and plotted as mean ± standard deviation.

### Cryo-EM grid preparation and data acquisition

In total, 10 mg/ml frozen aliquots of the mutant solution was spun down at 12,000*g* for 10 min, and the supernatant was diluted to a concentration of 2 mg/ml in protein storage buffer with 0.5 μM octyl glucoside, 1 mM TCEP (Fisher Scientific), and 1 mM ADP (Sigma Aldrich) or 1 mM ATPγS (Sigma Aldrich) or 1 mM CB-5083 (Cayman Chemical Company) depending on the experiment and incubated on ice before vitrification. Protein vitrification was achieved using the Leica GP2 plunge freezer on Quantifoil (R1.2/1.3, 200 or 300 mesh-Cu) holey carbon grids (Electron Microscopy Sciences). Firstly, coated grids were glow discharged at 25 mA for 15 s and placed on a pair of tweezers in the blotting chamber. Chamber temperature was set to 20 °C with 100% humidity. Three microliter of prepared protein solution was loaded on the grid. The grid was blotted for 6 s, followed by plunge-freezing in liquid ethane.

Frozen grids were stored in cryo grid boxes in liquid nitrogen until imaging. Digital micrographs of frozen hydrated protein particles were typically recorded at 190,000× magnification with defocus range between –1 and –2.5 μm with a total dose of 50 e^−^/Ǻ^2^ on either a 200 kV Glacios or 300 kV Krios (Thermo Fisher Scientific) transmission electron microscope fitted with a K3 (Gatan), Falcon3 or Falcon4 (Thermo Fisher Scientific) direct electron detector camera. Please refer to Table 1 for a full description of the cryo-EM data collection parameters.

**Table 1:** Statistics of Cryo-EM data collection, processing, and model refinement

	p97^R155H^-ADP	p97^R155H^-ATPγS	p97^R191Q^-ADP	p97^R191Q^-ATPγS	p97^A232E^-ADP	p97^A232E^-ATPγS	p97^E470D^-ADP	p97^E470D^-ATPγS	p97^D592N^-ADP	p97^D592N^-ATPγS	p97-CB-5083-ADP	p97-CB-5083-ATPγS
Cryo-EM data collection and processing												
Microscope	TF Titan Krios	TF Titan Krios	TF Titan Krios	TF Titan Krios	TF Titan Krios	TF Titan Krios	TF Titan Krios	TF Titan Krios	TF Titan Krios	TF Titan Krios	TF Titan Krios	TF Titan Krios
Magnification	105,000	105,000	105,000	105,000	105,000	105,000	105,000	105,000	105,000	150,000	150,000	150,000
Voltage (kV)	300	300	300	300	300	300	300	300	300	300	300	300
Detector	Gatan K3	Gatan K3	Gatan K3	Gatan K3	Gatan K3	Gatan K3	Gatan K3	Gatan K3	Gatan K3	Falcon 4	Falcon 4	Falcon 4
Dose rate (e^−^/pixel/s)	20	20	20	20	20	20	20	20	20	6	6	6
Total exposure (e^−^/Å^2^)	50	50	50	50	50	50	50	50	50	40	40	40
Defocus range (μm)	−1 to −2.5	−1 to −2.5	−1 to −2.5	−1 to −2.5	−1 to −2	−1 to −2	−1 to −2.5	−1 to −2.5	−1 to −2.5	−1 to −2.5	−1 to −2.5	−1 to −2.5
Pixel size (Å)	0.324	0.324	0.826	0.826	0.76	0.98	0.415	0.415	0.324	0.5	0.5	0.5
Micrographs collected	4410	5265	2000	4797	2569	640	4653	4810	6940	4464	3600	12,549
Symmetry imposed	C6	C6	C6	C6	C6	C6	C6	C6	C6	C6	D6	D6
Particles extracted/final	629,745/146,973	1,293,922/284,092	2,399,559/294,569	7,079,943/4,316,075	74,417/14,388	310,167/163,041	1,180,899/283,837	1,329,693/343,347	697,655/118,863	299,730/175,007	204,310/85,876	727,939/126,510
Map sharpening B-factor	126	109.5	131.2	153.7	116	126.2	106.2	94.2	104.3	77.1	116.8	148.1
Unmasked resolution at 0.5/0.143 FSC (Å)	3.9/3.2	2.9/2.6	3.6/3.1	3.1/2.8	3.3/2.7	3.2/2.7	3.5/2.9	3.0/2.5	3.7/3.0	2.7/2.2	3.2/2.6	4.2/3.5
Masked resolution at 0.5/0.143 FSC (Å)	3.8/3.1	2.8/2.5	3.5/3.1	3.1/2.7	3.2/2.7	3.2//2.6	3.4/2.7	2.8/2.5	3.6/2.9	2.6/2.2	3.0/2.4	4.2/3.4
Model building and validation												
Composition (#)												
Amino acids	4410	4422	4464	4422	4410	4422	4410	4368	4410	4368	8820	8736
Ligands ATPγS	0	12	0	12	0	12	0	12	0	12	0	12
ADP	12	0	12	0	12	0	12	0	12	0	12	0
MG	0	12	0	12	0	12	0	12	0	12	0	24
CB-5083	0	0	0	0	0	0	0	0	0	0	12	12
Bonds (RMSD)												
Length (Å)	0.004 (0)	0.004 (0)	0.04 (0)	0.004 (0)	0.005 (0)	0.004 (0)	0.002 (0)	0.004 (0)	0.003 (6)	0.004 (0)	0.004 (0)	0.004 (0)
Angles (°)	1.064 (0)	1.027 (0)	1.045 (0)	1.090 (12)	1.080 (12)	1.025 (0)	0.672 (42)	0.556(12)	0.744 (12)	0.601 (12)	0.826 (36)	1.107 (74)
Mean B-factors												
Amino Acids	193.88	146.33	170.66	182.41	153.36	189.3	147.89	139.4	172.28	128.87	136.46	216.93
Ligand	131.67	96.8	104.04	129.96	97.94	132.17	111.01	84.37	141.24	80.78	101.41	117.48
Ramachandran												
Favored (%)	94.85	93.84	91.6	92.45	95.96	94.41	96.03	95.86	95.37	95.67	94.4	93.22
Allowed (%)	4.88	6.16	8.13	7.55	3.76	5.32	3.83	3.87	4.63	4.05	5.32	6.23
Outliers (%)	0.27	0	0.27	0	0.27	0.27	0.14	0.28	0	0.28	0.27	0.55
Rotamer outliers (%)	0.32	0	0	0.03	0.03	0	0	0.19	0	0	0	0
Clash score	10.13	11.24	9.42	9.94	10.02	7.42	7.65	12.32	10.54	13.67	17.43	15.29
C-Beta outliers (%)	0	0	0	0	0	0	0	0	0	0	0	0.15
CaBLAM outliers (%)	3.44	3.16	5.3	4.94	3.44	4.39	3.58	3.33	3.99	3.33	3.71	5.28
CC (Mask)	0.77	0.82	0.81	0.79	0.8	0.79	0.78	0.82	0.79	0.84	0.82	0.79
MolProbity score	1.88	1.98	2	1.99	1.8	1.79	1.69	1.89	1.86	1.94	2.12	2.13

### Image processing

In general, all data processing was performed in cryoSPARC v.2.15 or v.3.0.1 (32) unless stated otherwise. Motion correction in patch mode, CTF estimation in patch mode, blob particle picking (using 160 Å blob as template), and particle extraction were performed on-the-fly in cryoSPARC. After preprocessing, particles were subjected to 2D classification and 3D heterogeneous refinement. Final 3D homogeneous refinement was done with per particle CTF estimation and aberration correction, C6 (for all the p97 mutants) or D6 (for p97 bound with CB5083 and ADP/ATPγS) symmetry applied. Resolution of the 3D map was determined according to the resulting Fourier Shell Correlation (FSC) curve at a cutoff criterion of FSC = 0.143 (33). Please refer to Table 1, for a full description and Figures S5–S16 for graphical representation of the cryo-EM structure determination and validation analysis.

### Model building and structure analysis

The p97^WT^ cryo-EM structures in the ADP (PDB ID: 5FTK) or ATPγS (PDB ID: 5FTN) bound states reported previously (5) were used to build initial models for the ADP- and ATPγS-bound mutants respectively. Using UCSF Chimera (34), the hexamer was fit into the map to get an initial structure for real-space refinement of the atomic model in PHENIX (35). The resulting atomic model was examined using the comprehensive validation tool in PHENIX. Figures were prepared using UCSF Chimera and ChimeraX (36).

## Data availability

The density maps and refined atomic models have been deposited in the Electron Microscopy Data Bank with accession numbers EMD-24518, 24519, 24522, 24523, 24524, 24525, 24526, 24528, 24529, 24530, 24531, and 24532 and in the Protein Data Bank with matching accession numbers of PDB-7RL6, 7RL7, 7RL9, 7RLA, 7RLB, 7RLC, 7RLD, 7RLF, 7RLG, 7RLH, 7RLI, and 7RLJ respectively, for R155H-ADP, R155H-ATPγS, R191Q-ADP, R191Q-ATPγS, A232E-ADP, A232E-ATPγS, E470D-ADP, E470D-ATPγS, D592N-ADP, D592N-ATPγS mutant p97, and for p97 bound to CB-5083-ADP and CB-5083-ATPγS. All other data are available from the corresponding authors upon request.

## Supporting information

This article contains supporting information (12, 15, 19, 37).

## Conflict of interest

S. S. is Founder and CEO of Gandeeva Therapeutics Inc, a drug discovery company based in Vancouver. All other authors declare that they have no conflicts of interest with the contents of this article.
