# Supplementary material for: AAA+ ATPase p97/VCP mutants and inhibitor binding disrupt inter-domain coupling and subsequent allosteric activation
Source: J Biol Chem. 2021 Sep 11;297(4):101187. doi: 10.1016/j.jbc.2021.101187 (PMC8517850; doi:10.1016/j.jbc.2021.101187)
Supplement: Figures S1–S16 and Table S1 [file mmc1.docx]

**Supporting Information**

**
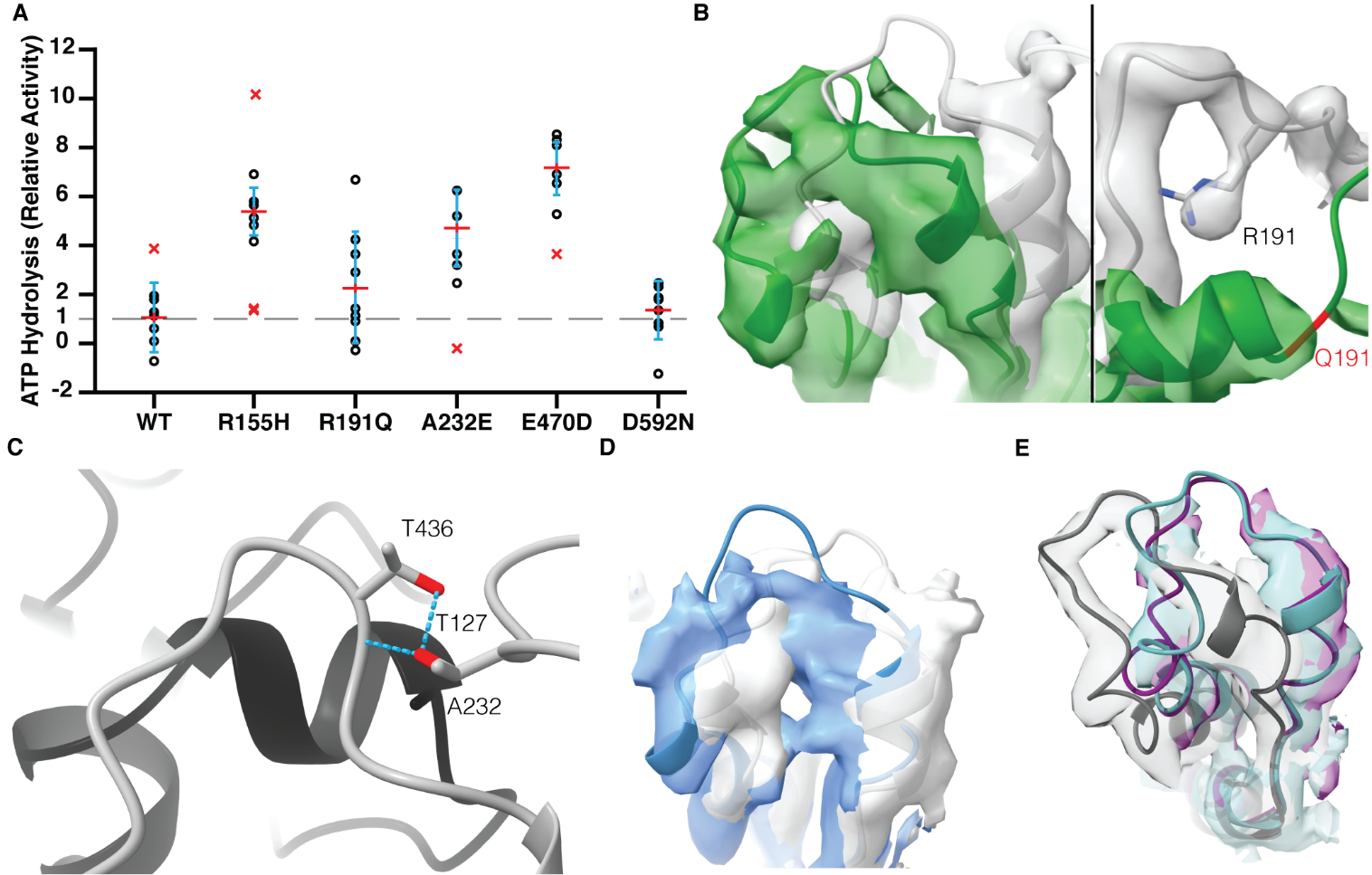
**

**Fig. S1: p97 Disease Mutants.**

**(A)** ATPase activity assay of BCA normalized concentrations of wild-type p97 and its mutants, comparable with previously published data (12,15,19). Data are from three inter-run replicates with three technical replicates within each run (*n*=9). Sample mean is indicated by the red line and error bars represent $\pm$SD, excluding outliers, defined as data outside of 1.5 inter-quartile range, indicated by x. **(B)** Overlaid ADP-bound densities of p97^WT^ (grey) and p97^R191Q^ (green) for fig.2 B+E, indicating the shift in mutant density relative to WT, sigma threshold values for mutant and WT in the left panel are 3.7 and 3.8 respectively and in the right panel 3.3 and 3.4. **(C)** Side view of A232 interacting residues between N and D1 domains in WT [PDB:5FTK], adjacent protomers labelled grey and black, tentative hydrogen bonds modelled in chimera, according to precise geometric constraints (37) based on labelled in cyan. **(D)** Overlaid ADP-bound densities of p97^WT^ (grey) and p97^A232E^ (green), indicating a similar shift to p97^R191Q^ in the loop region between K425 and L445 of the D1 domain in p97^A232E^ mutant density relative to WT, sigma threshold values for mutant and WT are 3.2 and 3.2 respectively. **(E)** Overlaid ATP$\gamma$S-bound densities of p97^WT^ (grey), p97^E470D^ (cyan) and p97^D592N^ (purple), indicating a shift in the loop region between K425 and L445 of the D1 domain in mutant density relative to WT, sigma threshold values for p97^E470D^, p97^D592N^ mutant and WT are 4.2, 4.2 and 4.2 respectively.


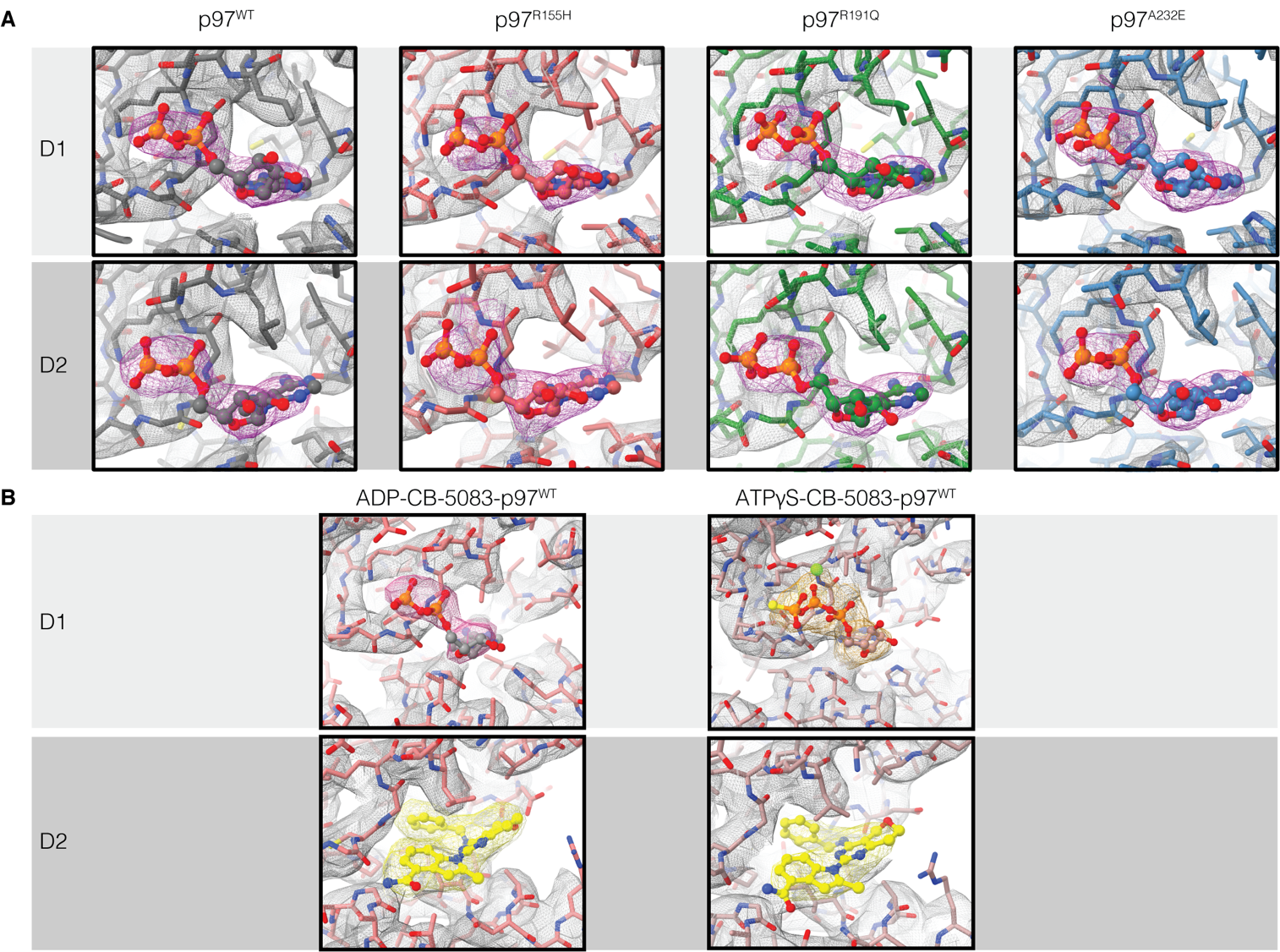


**Fig. S2: Nucleotide density in the D1 and D2 domains of the unsharpened cryo-EM maps of p97^WT^ and mutants.**

**(A)** Expanded view of nucleotide density in Fig. 2 models of p97^WT^ (grey), p97^R155H^ (red), p97^R191Q^ (green) and p97^A232E^ (blue) mutants, indicating the presence of ADP density (pink) in both the D1 and D2 domains. Average sigma threshold values for EM maps from left to right are 5.6, 2.9, 8.1 and 8.0. **(B)** Expanded view of nucleotide density in Fig. 4 models of p97^WT^ in the D1 and D2 domains of the unsharpened cryo-EM maps of CB5083 density (yellow) in p97 with either ADP (pink) or ATP$\gamma$S (orange) nucleotide. Average sigma threshold values for ADP-CB-5083-bound and ATP$\gamma$S-CB5083-bound EM maps are 6.4 and 4.6 respectively.


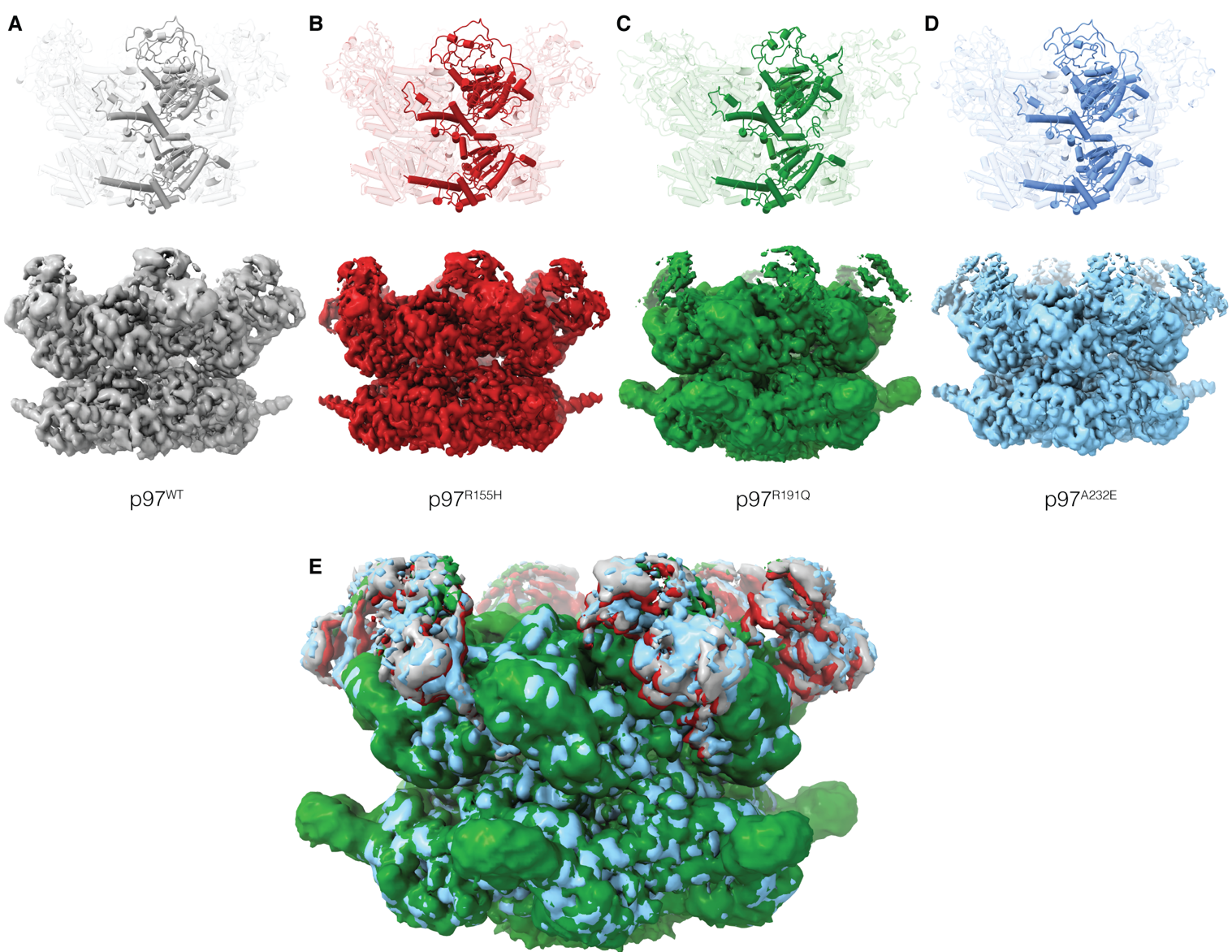


**Fig. S3: Cryo-EM Structures and their corresponding maps of ATP**$\boldsymbol{\gamma}$**S-Bound p97 and Disease Mutants R155H, R191Q and A232E.**

ATP$\gamma$S-bound p97 structures, indicate similar quarternary states, with the N-domain in the “Up” position. This suggests the mutants, **(B)** p97^R155H^, **(C)** p97^R191Q^ and **(D)** p97^A232E^ have little effect on the ATP$\gamma$S-bound quarternary structure of p97. **(E)** Overlay of EM densities highlighting similarity in structures. Sigma threshold values for structures are 3.2, 2.6, 0.7 and 2.2 respectively.

**
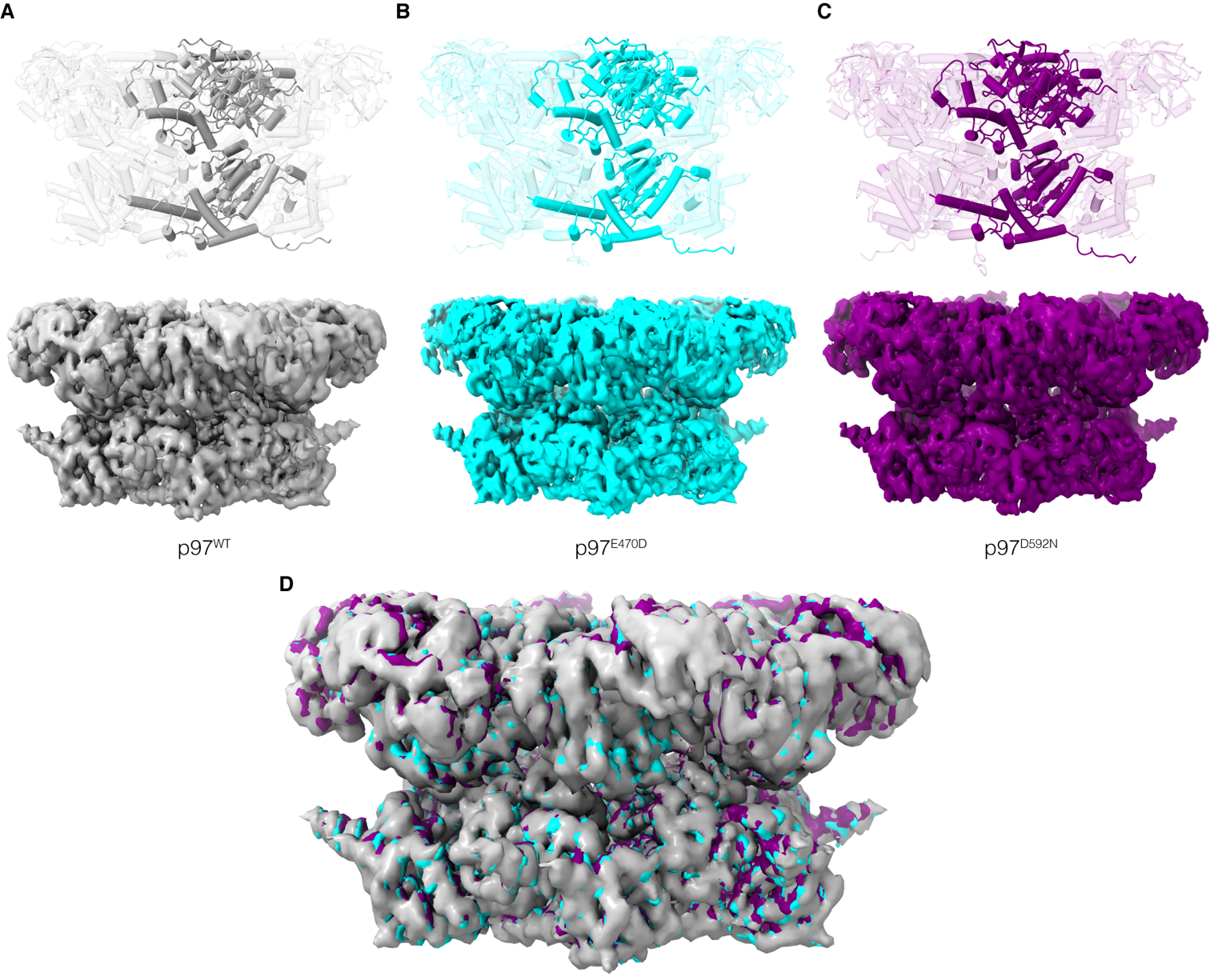
**

**Fig. S4: Cryo-EM Structures and their corresponding maps of ADP-Bound p97 and the Disease Mutants p97^E470D^ and p97^D592N^.**

ADP-bound p97 structures, indicate similar quarternary states, with the N-domain in the “Down” position. This suggests the mutants **(B)** p97^E470D^  and **(C)** p97^D592N^ have little effect on the ADP-bound quarternary structure of p97. **(D)** Overlay of EM densities highlighting similarity in structures. Sigma threshold values for structures are 2.7, 4.3, and 1.8 respectively.

**
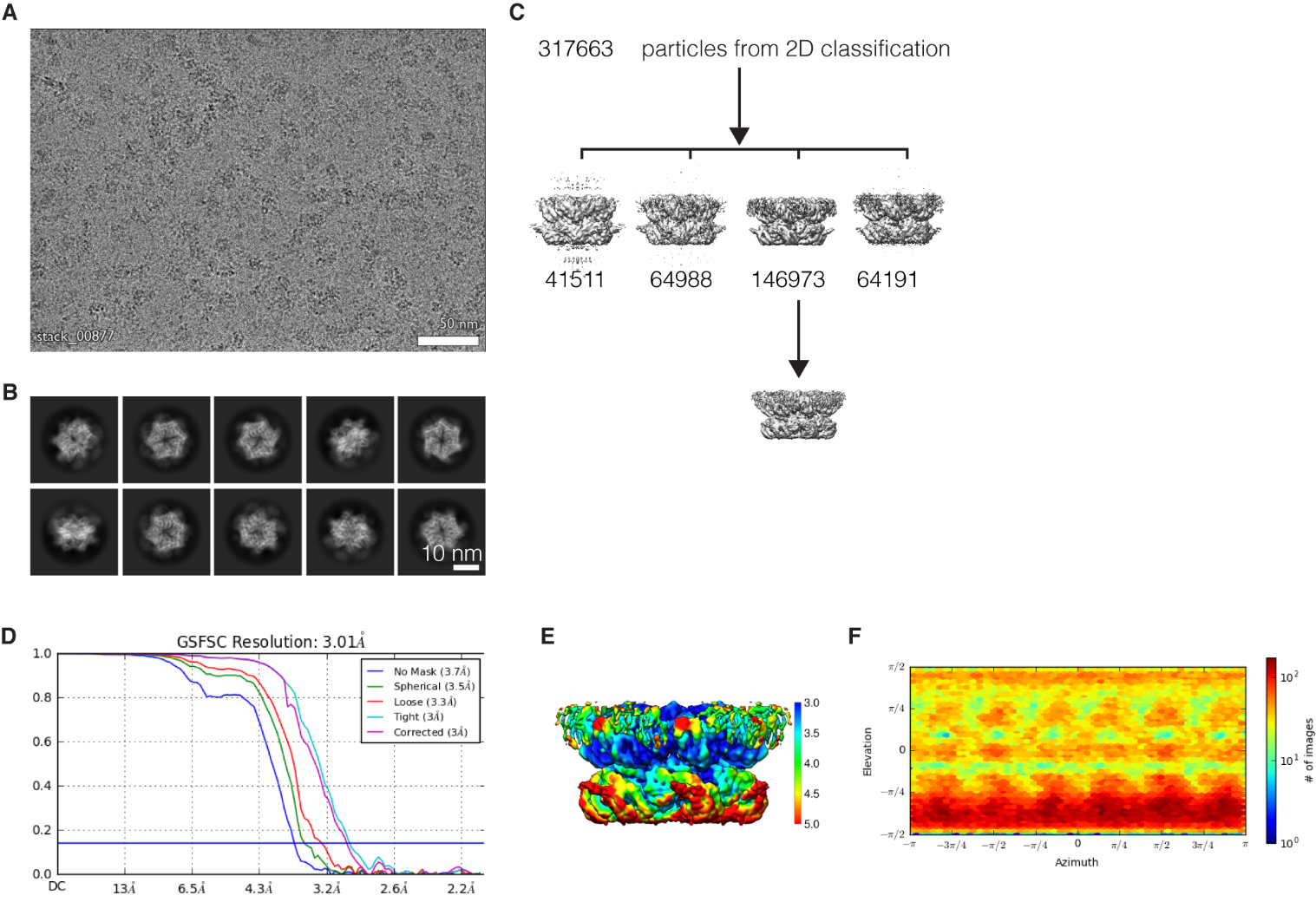
**

**Fig. S5: ADP-bound p97^R155H^.**

**(A)** A representative cryo-EM micrograph of ADP-bound p97^R155H^. **(B)** Representative 2D classes, showing multiple orientations. **(C)** Workflow of cryo-EM image processing. **(D)** FSC curves. **(E)** Cryo-EM side-view density of p97 mutants, colored according to local resolution and **(F)** Viewing direction distribution plot for mutant density.

**
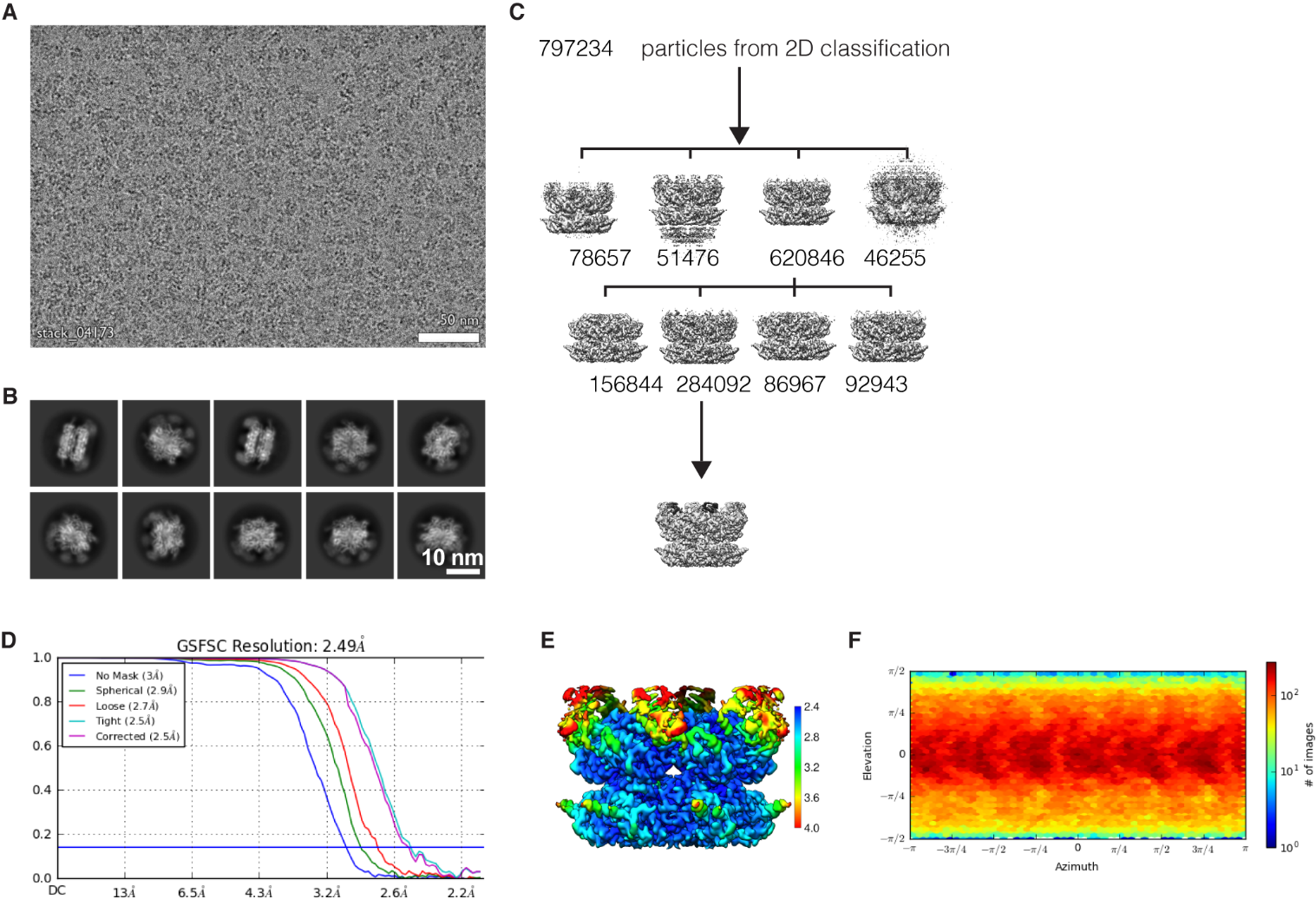
**

**Fig. S6: ATP**$\boldsymbol{\gamma}$**S-bound p97^R155H^.**

**(A)** A representative cryo-EM micrograph of ATP$\gamma$S-bound p97^R155H^. **(B)** Representative 2D classes, showing multiple orientations. **(C)** Workflow of cryo-EM image processing. **(D)** FSC curves. **(E)** Cryo-EM side-view density of p97 mutants, colored according to local resolution and **(F)** Viewing direction distribution plot for mutant density.

**
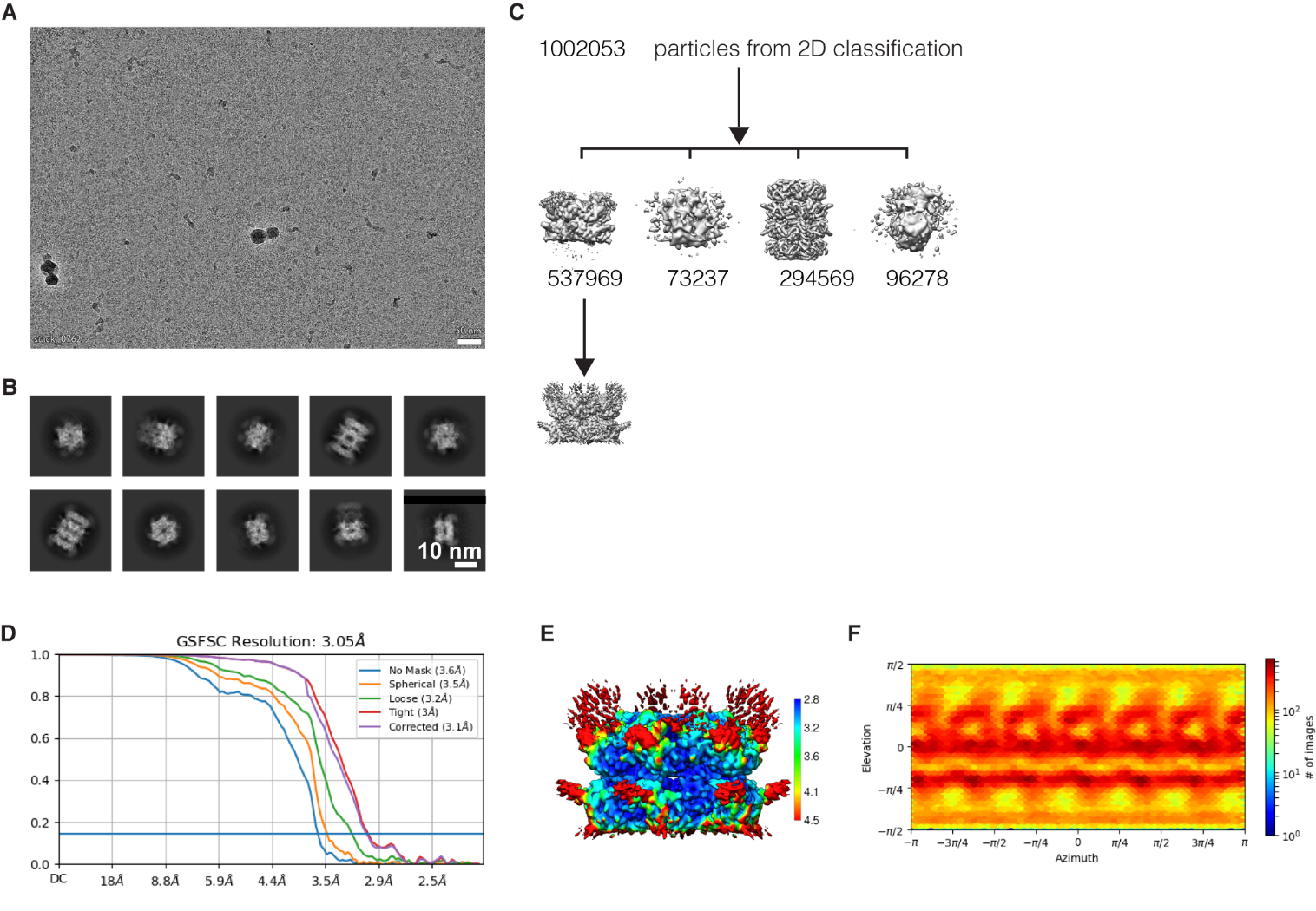
**

**Fig. S7: ADP-bound p97^R191Q^.**

**(A)** A representative cryo-EM micrograph of ADP-bound p97^R191Q^. **(B)** Representative 2D classes, showing multiple orientations. **(C)** Workflow of cryo-EM image processing. **(D)** FSC curves. **(E)** Cryo-EM side-view density of p97 mutants, colored according to local resolution and **(F)** Viewing direction distribution plot for mutant density.

**
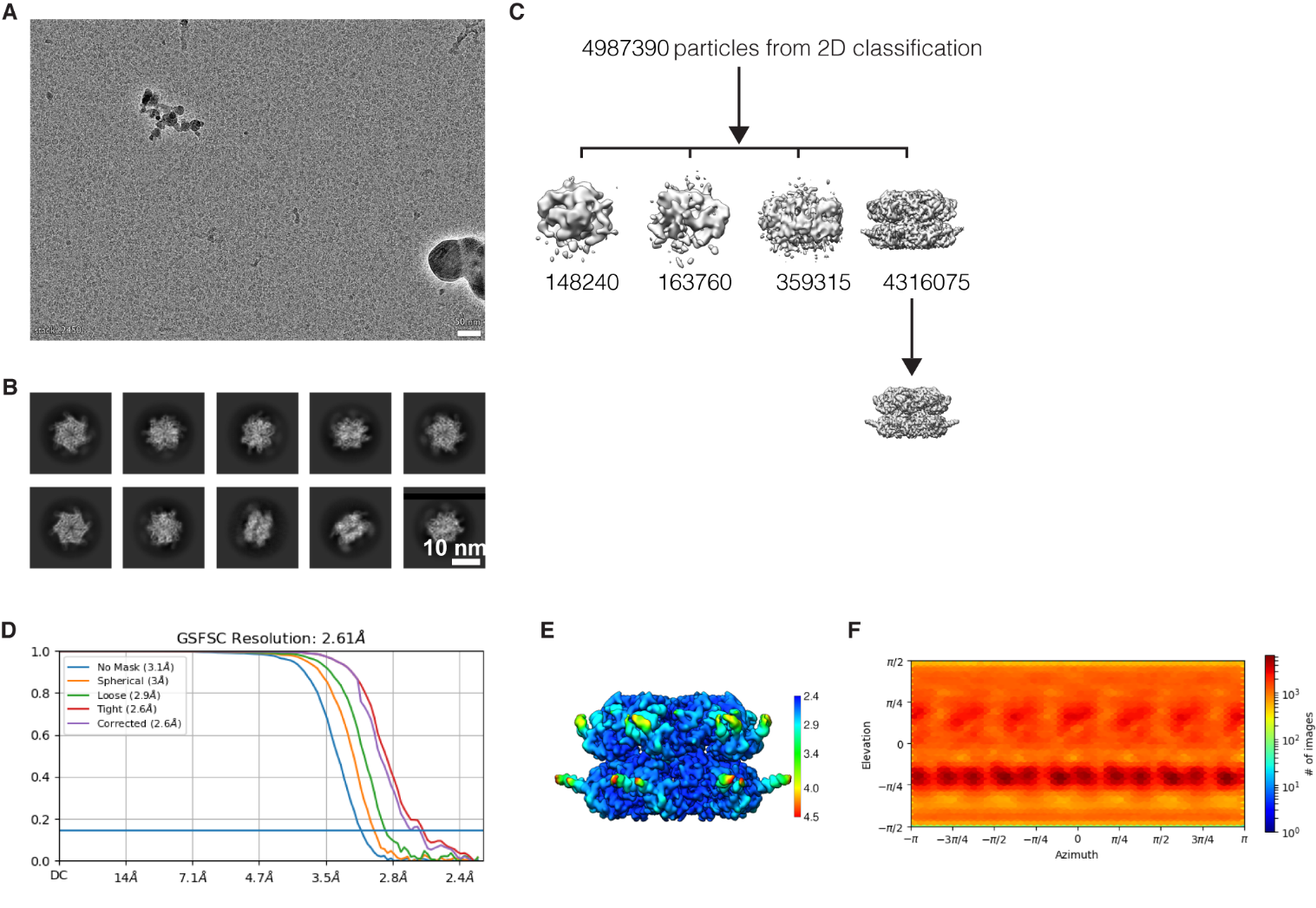
**

**Fig. S8: ATP**$\boldsymbol{\gamma}$**S-bound p97^R191Q^.**

**(A)** A representative cryo-EM micrograph of ATP$\gamma$S-bound p97^R191Q^. **(B)** Representative 2D classes, showing multiple orientations. **(C)** Workflow of cryo-EM image processing. **(D)** FSC curves. **(E)** Cryo-EM side-view density of p97 mutants, colored according to local resolution and **(F)** Viewing direction distribution plot for mutant density.

**
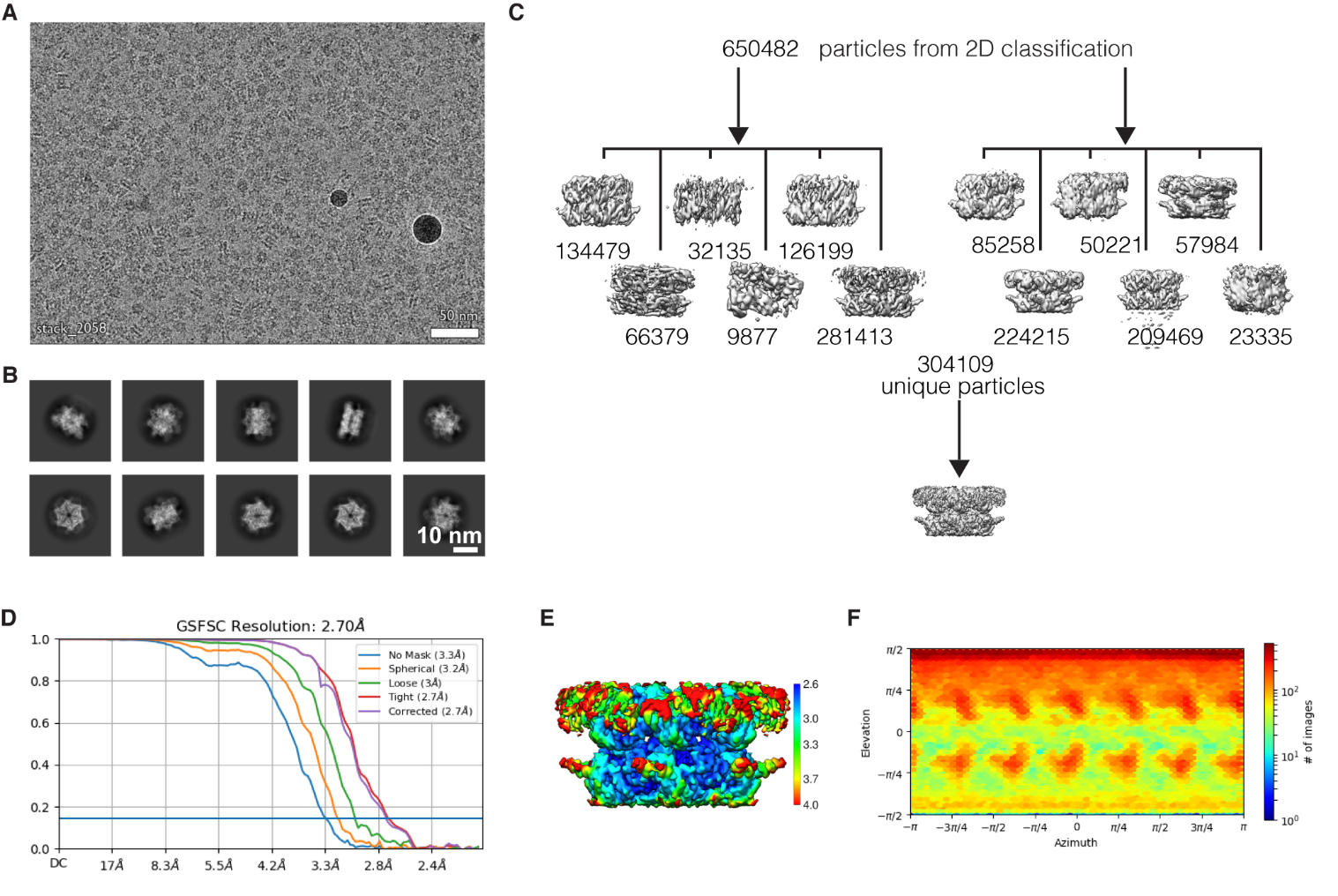
**

**Fig. S9: ADP-bound p97^A232E^.**

**(A)** A representative cryo-EM micrograph of ADP-bound p97^A232E^. **(B)** Representative 2D classes, showing multiple orientations. **(C)** Workflow of cryo-EM image processing. **(D)** FSC curves. **(E)** Cryo-EM side-view density of p97 mutants, colored according to local resolution and **(F)** Viewing direction distribution plot for mutant density.

**
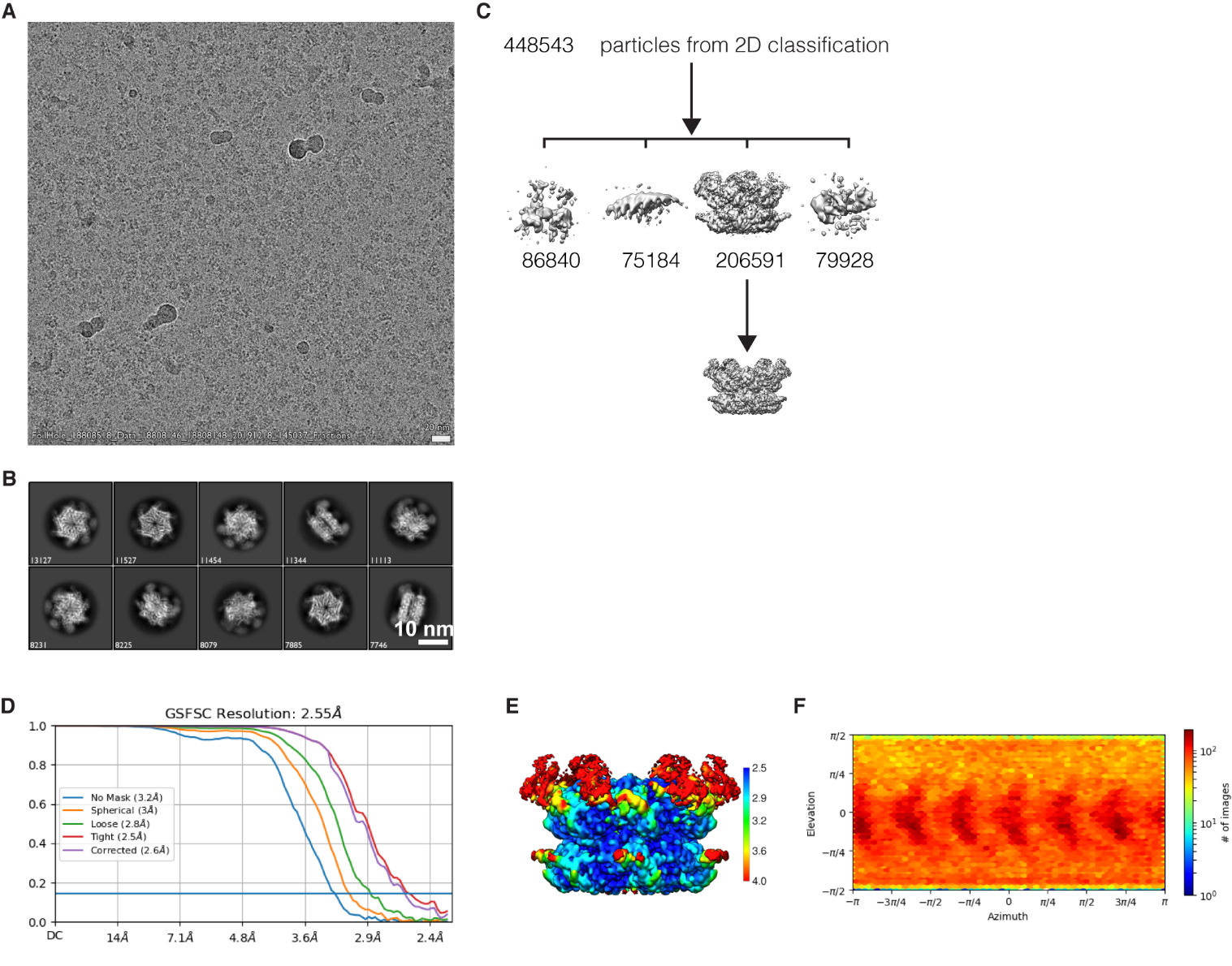
**

**Fig. S10: ATP**$\boldsymbol{\gamma}$**S-bound p97^A232E^.**

**(A)** A representative cryo-EM micrograph of ATP$\gamma$S-bound p97^A232E^. **(B)** Representative 2D classes, showing multiple orientations. **(C)** Workflow of cryo-EM image processing. **(D)** FSC curves. **(E)** Cryo-EM side-view density of p97 mutants, colored according to local resolution and **(F)** Viewing direction distribution plot for mutant density.

**
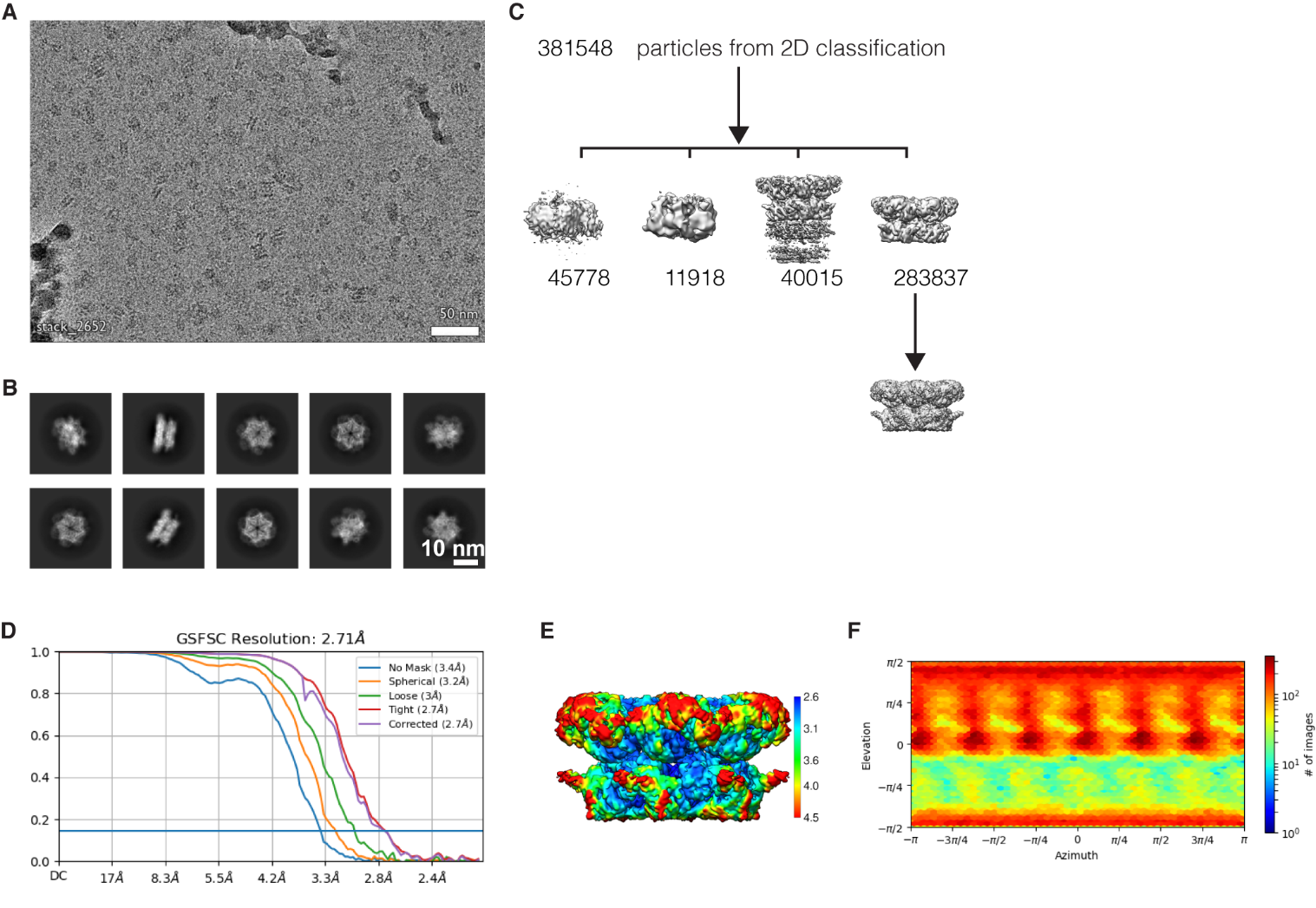
**

**Fig. S11: ADP-bound p97^E470D^.**

**(A)** A representative cryo-EM micrograph of ADP-bound p97^E470D^. **(B)** Representative 2D classes, showing multiple orientations. **(C)** Workflow of cryo-EM image processing. **(D)** FSC curves. **(E)** Cryo-EM side-view density of p97 mutants, colored according to local resolution and **(F)** Viewing direction distribution plot for mutant density.


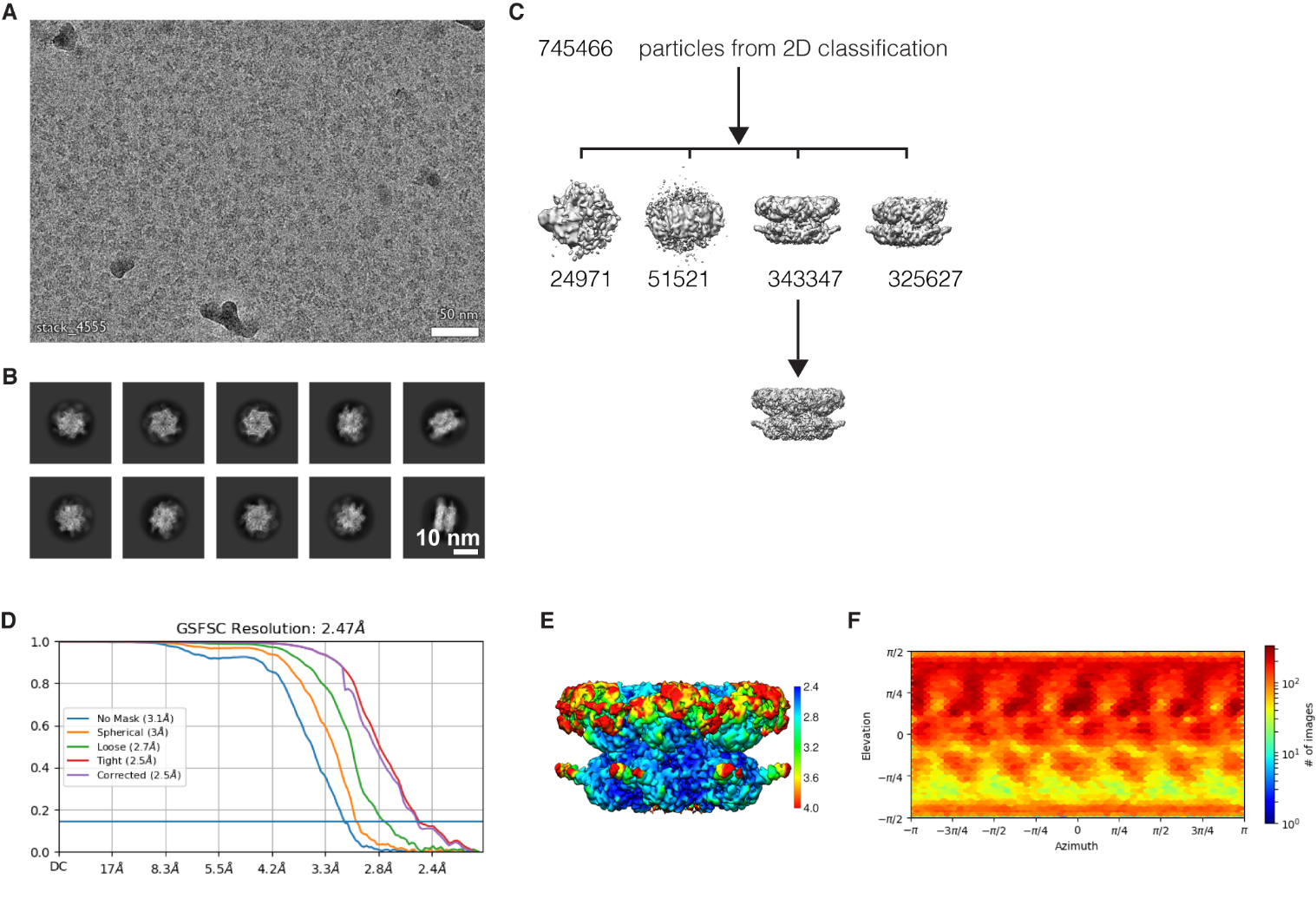


**Fig. S12: ATP**$\boldsymbol{\gamma}$**S-bound p97^E470D^.**

**(A)** A representative cryo-EM micrograph of ATP$\gamma$S-bound p97^E470D^. **(B)** Representative 2D classes, showing multiple orientations. **(C)** Workflow of cryo-EM image processing. **(D)** FSC curves. **(E)** Cryo-EM side-view density of p97 mutants, colored according to local resolution and **(F)** Viewing direction distribution plot for mutant density.


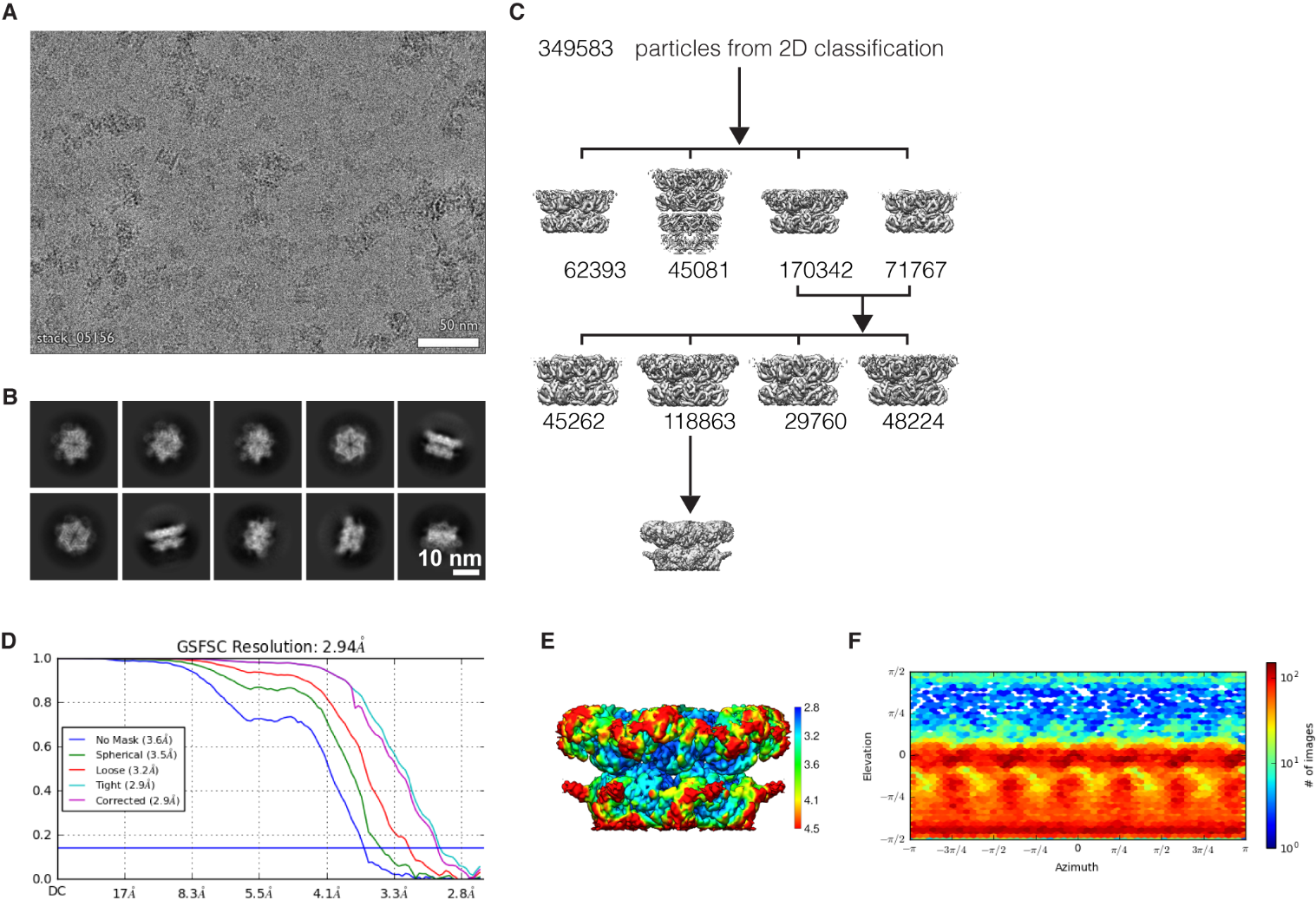


**Fig. S13: ADP-bound p97^D592N^.**

**(A)** A representative cryo-EM micrograph of ADP-bound p97^D592N^. **(B)** Representative 2D classes, showing multiple orientations. **(C)** Workflow of cryo-EM image processing. **(D)** FSC curves. **(E)** Cryo-EM side-view density of p97 mutants, colored according to local resolution and **(F)** Viewing direction distribution plot for mutant density.

**
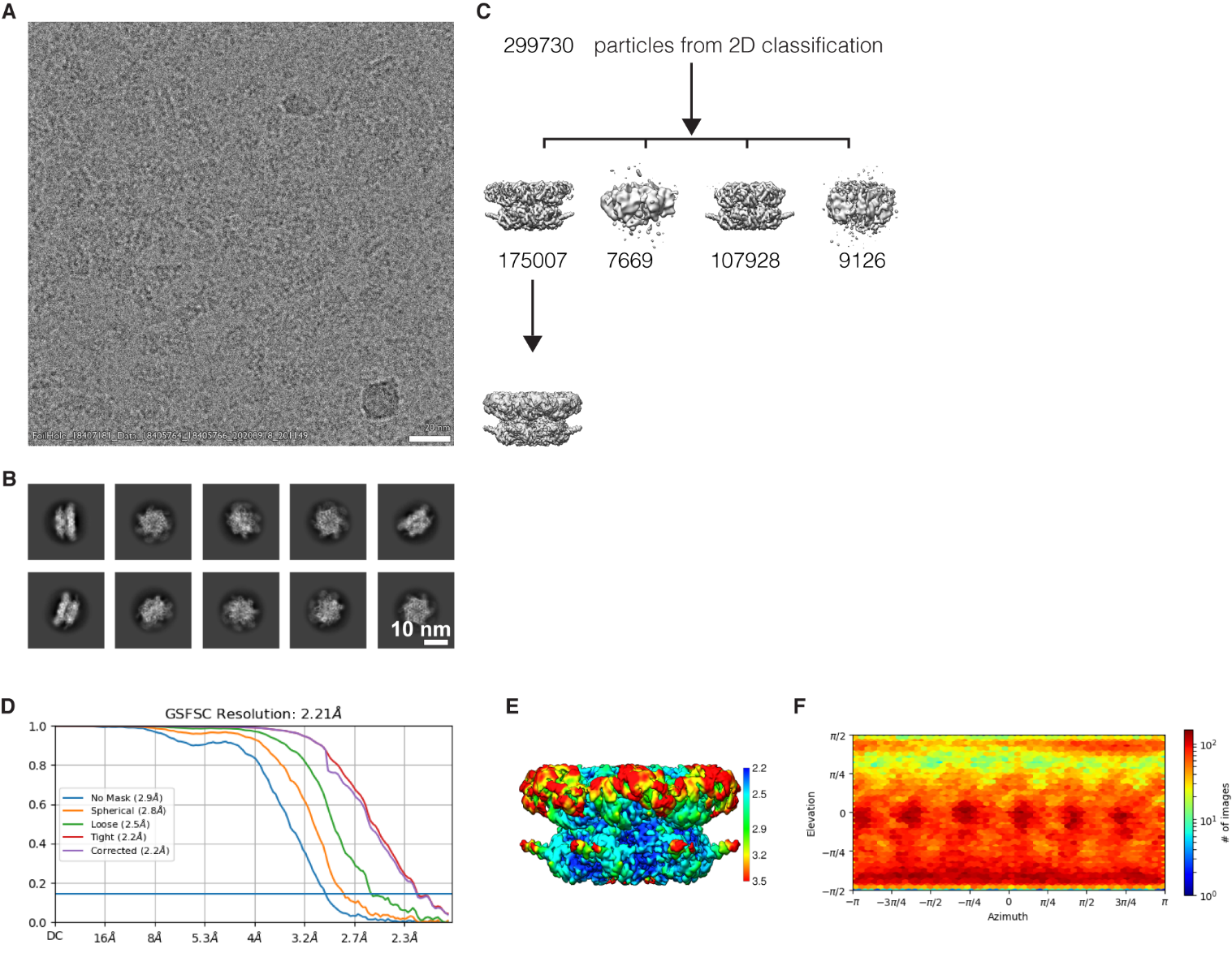
**

**Fig. S14: ATP**$\boldsymbol{\gamma}$**S-bound p97^D592N^.**

**(A)** A representative cryo-EM micrograph of ATP$\gamma$S-bound p97^D592N^. **(B)** Representative 2D classes, showing multiple orientations. **(C)** Workflow of cryo-EM image processing. **(D)** FSC curves. **(E)** Cryo-EM side-view density of p97 mutants, colored according to local resolution and **(F)** Viewing direction distribution plot for mutant density.


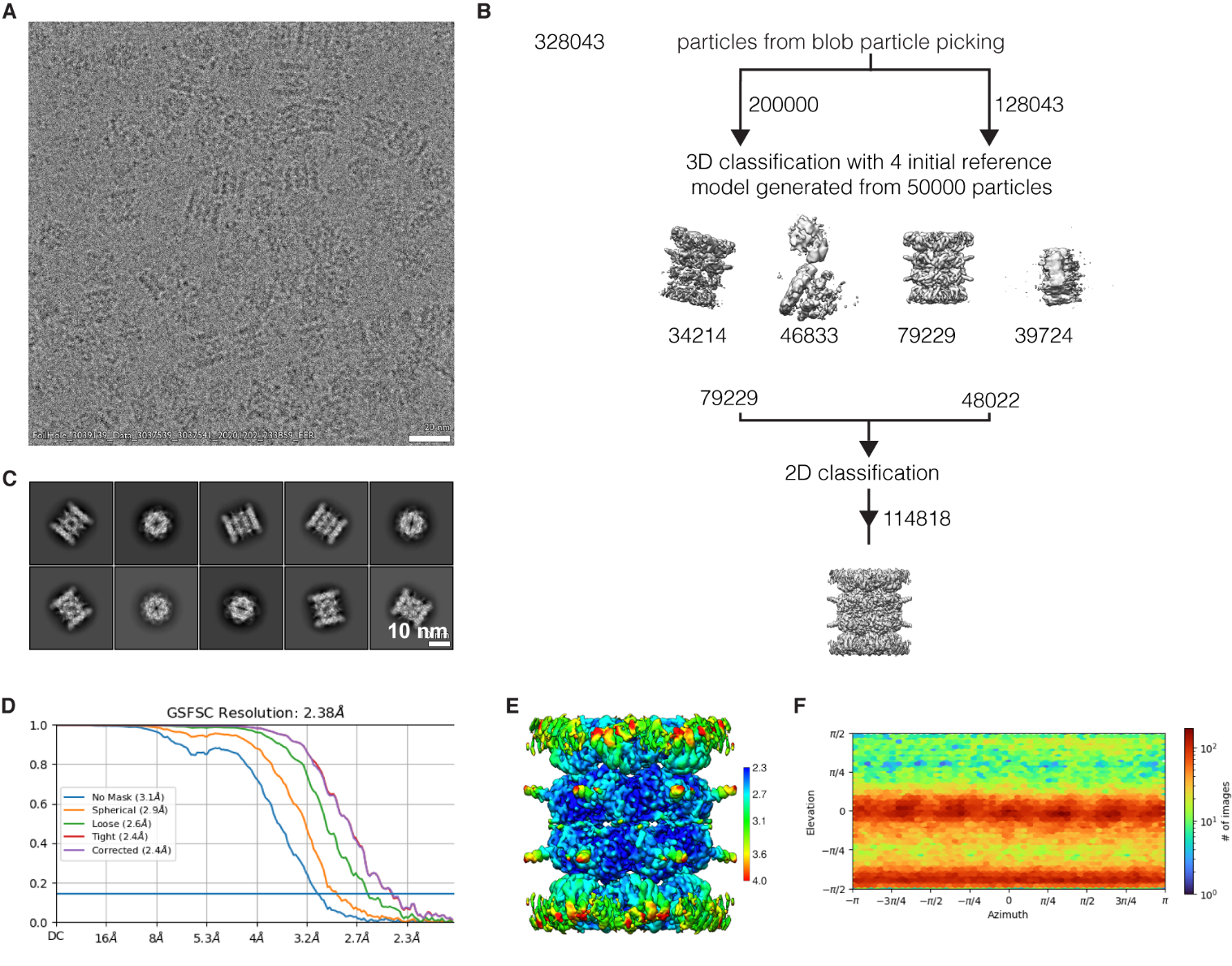


**Fig. S15: CB-5083-bound ADP-p97.**

**(A)** A representative cryo-EM micrograph of D1-ADP-D2-CB-5083-bound p97^WT^. **(B)** Representative 2D classes, showing multiple orientations. **(C)** Workflow of cryo-EM image processing. **(D)** FSC curves. **(E)** Cryo-EM side-view density of p97, colored according to local resolution and **(F)** Viewing direction distribution plot for mutant density.


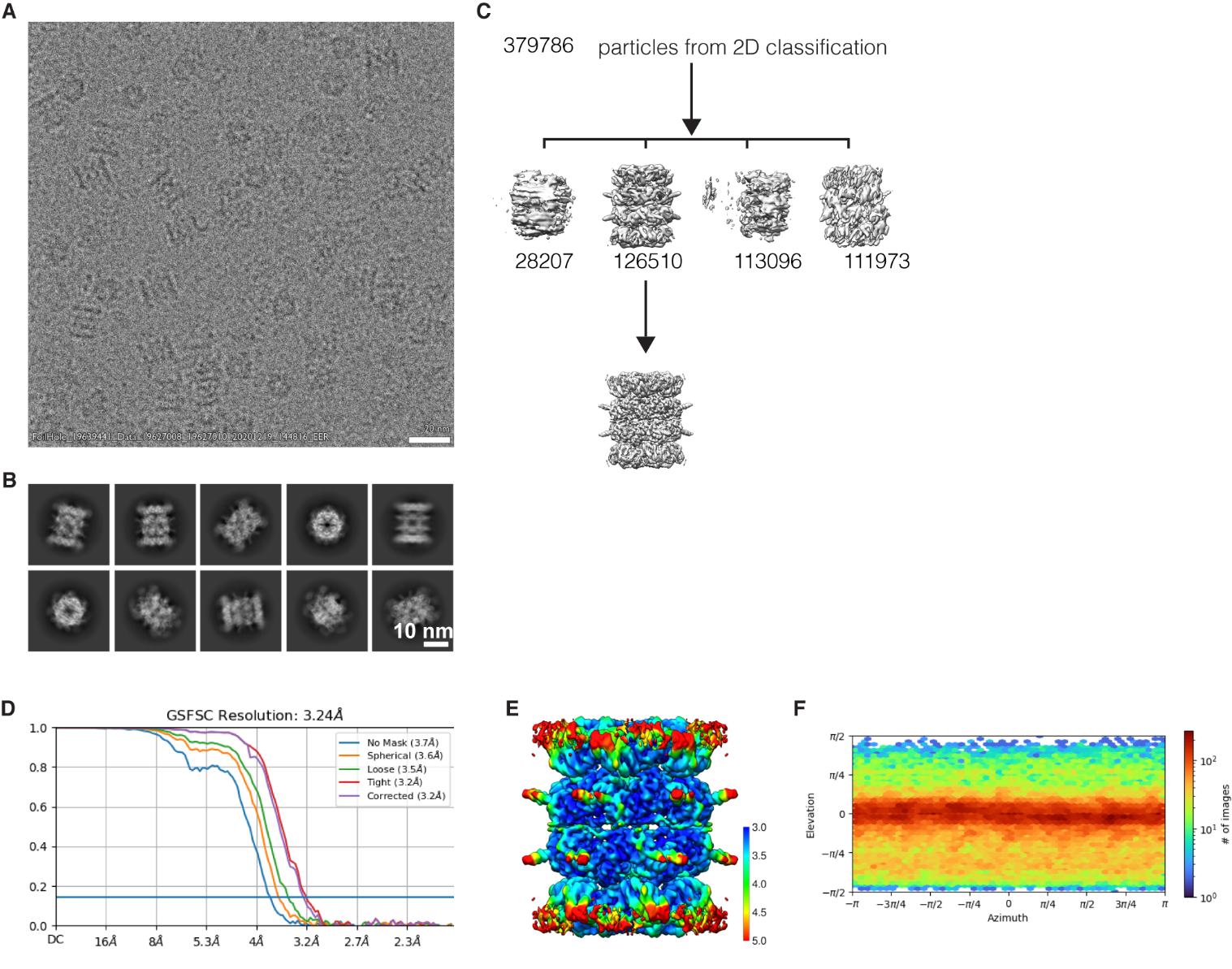


**Fig. S16: CB-5083-bound ATP**$\boldsymbol{\gamma}$**S-p97.(A)** A representative cryo-EM micrograph of D1-ATP$\gamma$S-D2-CB-5083-bound p97^WT^. **(B)** Representative 2D classes, showing multiple orientations. **(C)** Workflow of cryo-EM image processing. **(D)** FSC curves. **(E)** Cryo-EM side-view density of p97 mutants, colored according to local resolution and **(F)** Viewing direction distribution plot for mutant density.

**SUPPLEMENTAL TABLES**

**Table S1. Oligonucleotides Synthesized by ThermoFisher for this Study, Related to Key Resources Table.**

| Name and Sequence of the DNA Oligo | Identifier |
| --- | --- |
| PCR oligonucleotide primer - R155H Forward - 5'-TTTCTGGTTCACGGTGGCATG-3' | N/A |
| PCR oligonucleotide primer - R155H Reverse - 5'-GATGTCACCCTTACGGATC-3' | N/A |
| PCR oligonucleotide primer - R191Q Forward - 5'-CCGATTAAACAAGAAGATGAAGAAGAGTCC-3' | N/A |
| PCR oligonucleotide primer - R191Q Reverse - 5'-CTCACCCTCGCAGTGAAT-3' | N/A |
| PCR oligonucleotide primer - A232E Forward - 5'-CTGTTCAAAGAGATTGGCGTGAAGCCGCC-3' | N/A |
| PCR oligonucleotide primer - A232E Reverse - 5'-CAACCTGGCGGACCATAA-3' | N/A |
| PCR oligonucleotide primer - E470D Forward - 5'-TTGTTGACGTGCCGCAAG-3' | N/A |
| PCR oligonucleotide primer - E470D Reverse - 5'-CAGTTTCGCGCAGGGC-3' | N/A |
| PCR oligonucleotide primer - D592N Forward - 5'-CAACATTGGTAACGGTGGCGGCG-3' | N/A |
| PCR oligonucleotide primer - D592N Reverse - 5'-CCGCCACGTGCCTTGGCG-3' | N/A |
| Sanger Sequencing primer - p97F1 Forward - 5'-GCGACGAGAAAATTCGCATGA-3' | N/A |
| Sanger Sequencing primer - p97R1 Reverse - 5'-GGCAGCTCAACCATTTCTTTAAT-3' | N/A |
| Sanger Sequencing primer - p97F2 Forward - 5'-GCTGGCAGTTACGATGGATGA -3' | N/A |
| Sanger Sequencing primer - p97R2 Forward - 5'-GCTCACGACGGATCTCGCT-3' | N/A |
